# ALACEN: A Holistic Herbaceous Biomass Fractionation Process Attaining a Xylose-Rich Stream for Direct Microbial Conversion to Bioplastics

**DOI:** 10.1021/acssuschemeng.3c08414

**Published:** 2024-05-08

**Authors:** Salvador Bertran-Llorens, Wen Zhou, Martín A. Palazzolo, Dana l. Colpa, Gert-Jan W. Euverink, Janneke Krooneman, Peter J. Deuss

**Affiliations:** † Green Chemical Reaction Engineering, Engineering and Technology Institute Groningen (ENTEG), University of Groningen, Nijenborgh 4, Groningen 9747 AG, The Netherlands; ‡ Products and Processes for Biotechnology, Engineering and Technology Institute Groningen (ENTEG), Faculty of Science and Engineering, University of Groningen, Nijenborgh 4, Groningen 9747 AG, The Netherlands; § Instituto Interdisciplinario de Ciencias Básicas (ICB, UNCuyo-CONICET), Padre Jorge Contreras 1300, Mendoza 5500, Argentina; ∥ Instituto de Investigaciones en Tecnología Química (INTEQUI), FQByF, Universidad Nacional de San Luis, CONICET, Almirante Brown 1455, San Luis 5700, Argentina; ⊥ Bioconversion and Fermentation Technology, Research Centre Biobased Economy, Hanze University of Applied Sciences, Zernikeplein 11, Groningen 9747 AS, The Netherlands

**Keywords:** lignocellulose fractionation, PHB, lignin, ferulic acid, dilute acid pretreatment, alkaline pretreatment, enzymatic saccharification

## Abstract

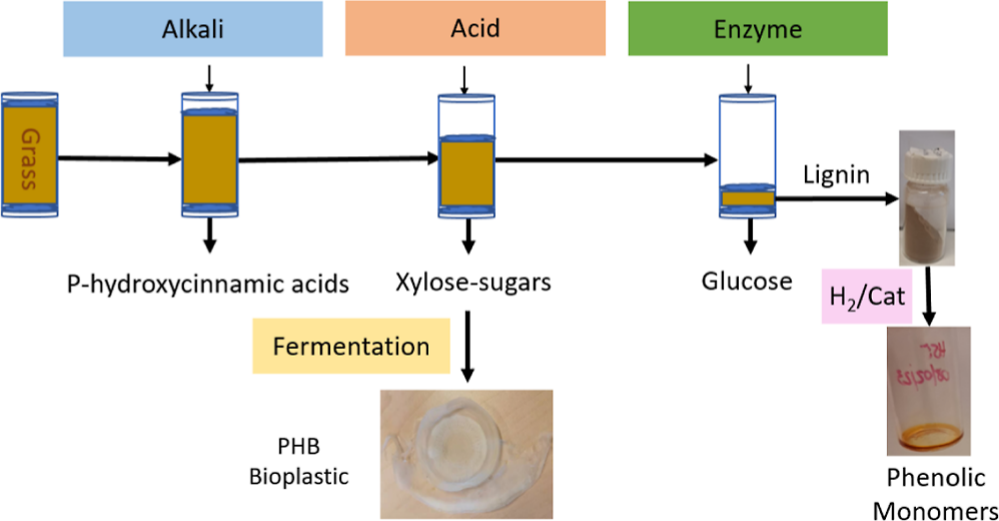

Lignocellulose biorefining is a promising technology for the sustainable production of chemicals and biopolymers. Usually, when one component is focused on, the chemical nature and yield of the others are compromised. Thus, one of the bottlenecks in biomass biorefining is harnessing the maximum value from all of the lignocellulosic components. Here, we describe a mild stepwise process in a flow-through setup leading to separate flow-out streams containing cinnamic acid derivatives, glucose, xylose, and lignin as the main components from different herbaceous sources. The proposed process shows that minimal degradation of the individual components and conservation of their natural structure are possible. Under optimized conditions, the following fractions are produced from wheat straw based on their respective contents in the feed by the ALkaline ACid ENzyme process: (i) 78% ferulic acid from a mild **AL**kali step, (ii) 51% monomeric xylose free of fermentation inhibitors by mild **AC**idic treatment, (iii) 82% glucose from **EN**zymatic degradation of cellulose, and (iv) 55% native-like lignin. The benefits of using the flow-through setup are demonstrated. The retention of the lignin aryl ether structure was confirmed by HSQC NMR, and this allowed monomers to form from hydrogenolysis. More importantly, the crude xylose-rich fraction was shown to be suitable for producing polyhydroxybutyrate bioplastics. The direct use of the xylose-rich fraction by means of the thermophilic bacteria *Schlegelella thermodepolymerans* matched 91% of the PHA produced with commercial pure xylose, achieving 138.6 mg_PHA_/g_xylose_. Overall, the ALACEN fractionation method allows for a holistic valorization of the principal components of herbaceous biomasses.

## Abstract 2

ALACEN is a new process that allows for obtaining valuable products like bioplastics and phenolic monomers from biomass.

## Introduction

The petroleum dependency of our current society as a major carbon source is deeply impacting our environment and our lives. In recent years, lignocellulosic biomass (LBM) has emerged as an alternative for producing biobased chemicals, biofuels, biopolymers, and other valuable products.^1^ LBM is abundant and well distributed globally as a leftover from industrial or agricultural activities. The production of biobased polymers and polymer precursors from LBM is projected to be one of the critical elements for the transition to a carbon-neutral society.^2^

Polyhydroxyalkanoates (PHAs) are naturally occurring polymers produced by bacteria as energy storage and in response to environmental stress.^3^ The bacterial granule accumulations are made out of bifunctional chemical blocks, with the most common being 3-hydroxybutyrate. These are combined to form a biodegradable polymer that has comparable strength and melting temperature to polypropylene but different mechanical properties. Such properties can be tuned by the introduction of cofactors to produce copolymers^4,5^ or by introducing natural fibers,^6^ thus representing a biodegradable alternative to petrol-based plastics. The main drawback of such PHAs is the high production cost compared to petrol-based plastics lying in the range between 2.2 and 5 €/kg,^7^ which stresses the need to search for versatile residual carbon sources like side streams from different feedstock biorefineries.^8^

Among the different LBM from agro-waste sources, the herbaceous ones (*Poaceae* family) are attractive due to (i) the high carbohydrate fraction (25–40% cellulose and 25–50% hemicellulose), (ii) they derive from fast-growing crops cultivated annually worldwide, and (iii) their relatively high amounts of readily extractable valuable compounds like ferulic acid (FA) and coumaric acid (CA).^9^

**Figure 1:**
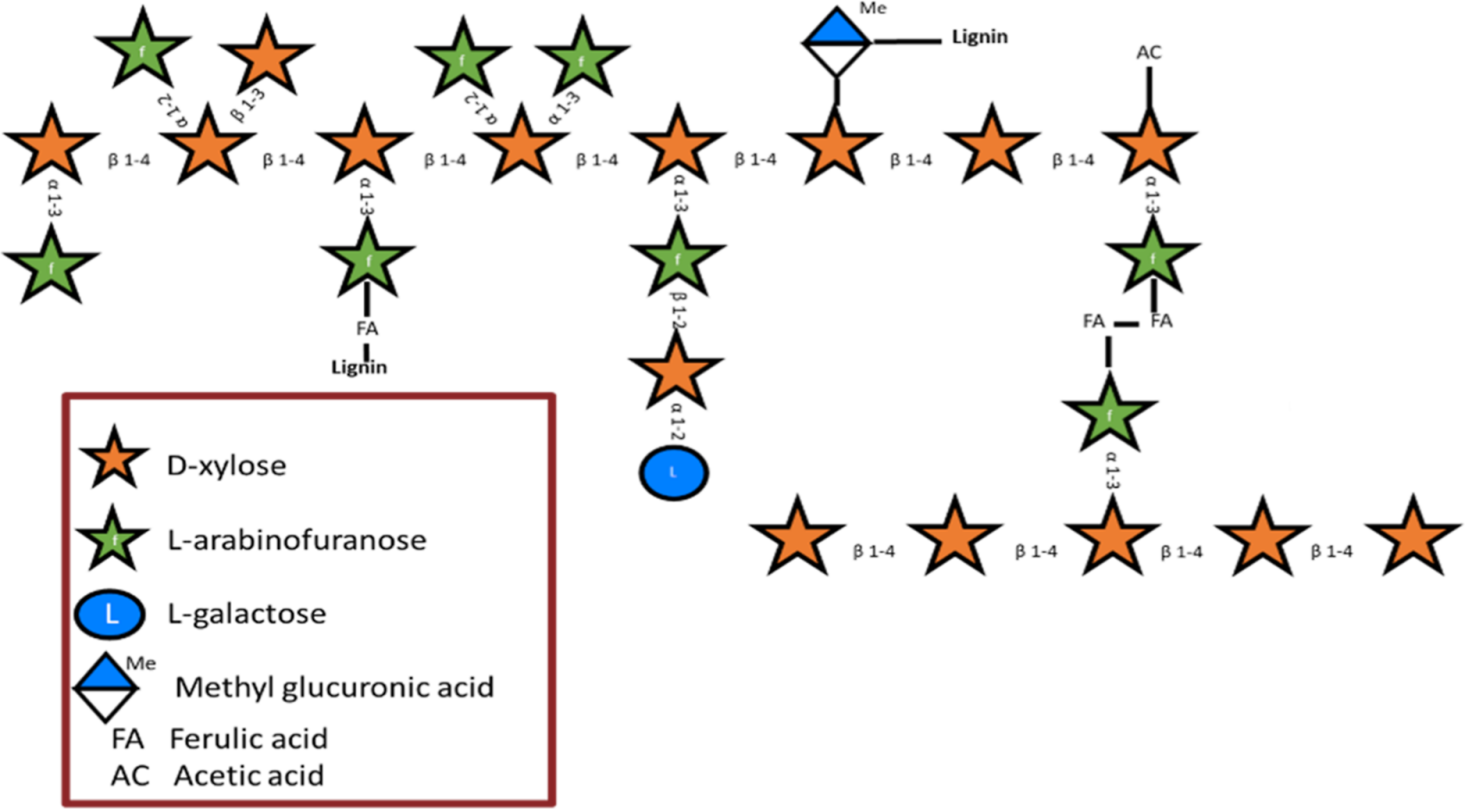
GAX schematized structure, showing the interlinkage between xylan backbones and xylan-lignin via FA as well as the interlinkage of methyl glucuronic acid and lignin.

For most of the herbaceous biomasses, hemicellulose is composed of complex glucuronoarabinoxylan (GAX) containing a xylose backbone linked via β-D-(1 → 4) bonds and decorated with different C5 (mainly arabinose) and C6 (mainly glucuronic acid and galactose) sugars, as well as other groups such as hydroxycinnamoyl and acetyl (Figure 1). Specifically, FA is of particular interest due to its key role in the interlinkage of the xylan backbone via ester linkages^10^ and its value due to its high demand and market price (predicted to reach 90 M US$ globally by 2026).^11^ On top of the added value, some microorganisms were shown to be particularly sensitive to FA, thus defaulting the subsequent fermentation of xylose to valuable products.^12^

Xylose is an important underutilized fermentation carbon source compared to cellulose-derived glucose.^13^ The main reason for this is that many bacteria and yeast prefer C6 sugars over C5 sugars and usually display catabolic repression to utilize only one sugar.^14^ The utilization of thermophilic bacteria with high PHA production from underutilized products like xylose offers a promising biorefinery opportunity.^15^ Thermophilic bacteria are industrially relevant due to fewer problems with contamination of the culture as well as their easy blend-in in coprocesses like fermentation and enzymatic cellulose hydrolysis due to similar optimum temperatures (50 °C).^16,17^

**Figure 2:**
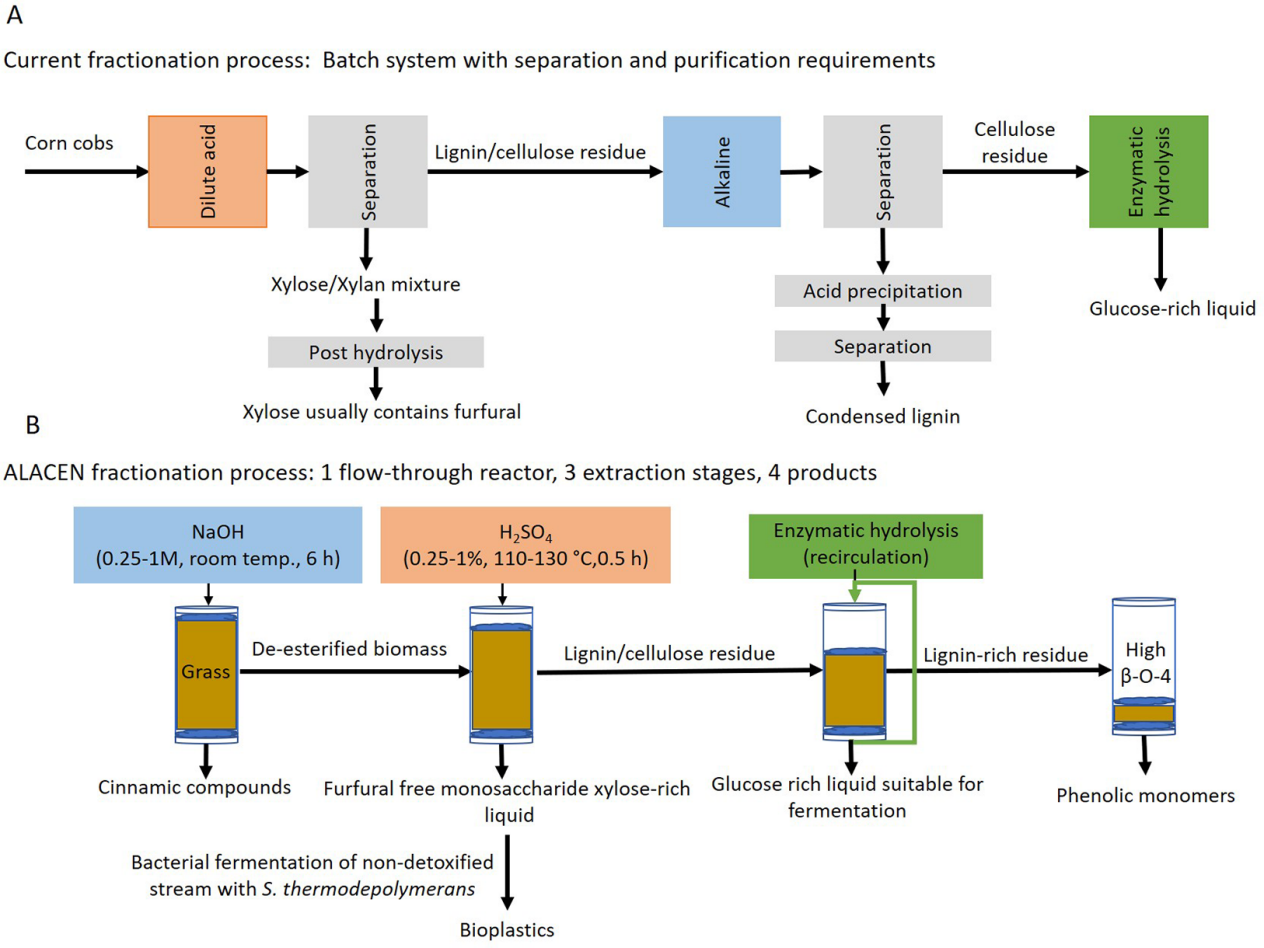
Graphic description of (A) an advanced current stepwise fractionation process^20^ and (B) the proposed flow-through ALACEN process in which all indicated steps are carried out in a single reactor.

Most of the current biorefinery processes focus on recovering cellulose as fibers or as monosaccharides for fermentation. The remainder of the components are typically degraded significantly, reducing their valorization potential or adding to the need to do a purification step to remove the degraded products (Figure 2a).^18,19^

Thus, for the development of industry-relevant biorefining processes based on herbaceous biomass sources, it is important to investigate fractionation approaches that allow for gaining maximum value out of all of the different lignocellulose components.

In this research, a sequential flow-through fractionation process for herbaceous biomasses is introduced that combines mild ALkaline and mild dilute ACid pretreatments with ENzymatic saccharification (ALACEN), with the aim of attaining four valuable fractions (Figure 2b). Each treatment has a specific function: mild diluted alkaline treatment is intended to selectively remove esterified compounds like FA, dilute acid treatment is expected to solubilize most of the xylan without the formation of sugar degradation products such as furans, and the enzymatic hydrolysis aims to maximize the amount of monomeric glucose from the cellulose residue.

Several technologies have emerged in the past few years in the context of holistic approaches to biomass fractionation such as deep eutectic solvents, acid hydrotropic fractionation, or the DMR method.^21−28^ The most related approach is the combination of hemicellulose solubilization via dilute acid followed by lignin dissolution via alkaline treatments to enhance enzymatic cellulose removal.^29−31^ One of the main problems of these technologies is the condensation of lignin during alkaline solubilization^30^ and the production of sugar dehydration products in the hemicellulose-derived streams due to the high temperature and acid concentration needed to extract the hemicellulose-derived sugars.^32,33^ The latter led to the use of dilute acid loadings, but this failed to produce complete hydrolysis of xylan. The produced hydrolysates with complex xylooligosaccharides generate the need to perform a post-hydrolysis reaction to break the oligosaccharides into monosaccharides and do not solve the issue of coextracting FA among other compounds, which can act as inhibitors.^34−37^ Thus, there is still a need for developing fractionation strategies that allow for the direct utilization of crude biorefinery xylose-rich streams for the production of valuable products like PHA.^17^

One way to improve the current status is the usage of a flow-through extraction; these systems present several advantages compared to conventional batch systems. One is that biomass only needs to be loaded once, and all different fractions can be attained by simply changing the mobile liquid phase. Another key advantage is the continuous removal of extracted dissolved material, limiting elongated exposure to the reaction conditions and thus preventing undesired secondary reactions such as carbohydrate dehydration to furans, allowing for streams low in fermentation inhibitors.^38,39^ However, one of the main drawbacks of such technologies is the associated use of higher quantities of solvent. To avoid this in the described process, typical solid-to-liquid ratios used in batch reactions were applied. Moreover, in the ALACEN process, the specificity of the reactions was optimized using mild conditions (*T* < 130 °C, H_2_SO_4,_ and NaOH <1%) to avoid the formation of furfural and the degradation of other components like lignin (Figure 1).

To showcase the advantages of the ALACEN process, the crude neutralized xylose-rich stream was used as a direct carbon source for the production of PHA by the thermophilic bacteria *Schlegelella thermodepolymerans.* Furthermore, the lignin conservation was verified by structural analysis and application in catalytic reductive hydrogenolysis, and finally, the cellulose was tested for enzymatic saccharification.

## Experimental Section

### Materials

All of the chemicals used were purchased from Sigma-Aldrich (St. Louis, MO, USA) unless otherwise noted. Media components and salts were obtained from Merck (Kenilworth, NJ, USA), Difco (Becton Dickinson Company, Franklin Lakes, NJ, USA), Biosolve Chimie (Dieuze, France), Becton Dickinson Company (Franklin Lakes, NJ, USA), and BOOM (Meppel, The Netherlands). Beechwood, reeds, corn cobs, and corn leaf were acquired from local sources, and corn fiber was generously supplied by ADM (Decatur, IL, USA). Wheat straw was kindly provided by Idaho National Laboratory (Idaho, USA), and sugar cane bagasse was kindly provided by Dr. Genebaldo S. F. Neto and was the residue of ethanol and sugar production from sugar cane in northwest São Paulo State, Brazil.

### Biomass Preparation

All the biomasses were ground in a Perten lab mill 3303 and sieved to a particle size between 0.6 and 1 mm followed by a dewaxing step as previously reported.^40^ The extractives were discarded, and the biomass was dried under reduced pressure on a rotary evaporator and subsequently in a vacuum oven at 50 °C overnight.

### ALACEN Fractionation

The dewaxed biomass was loaded in the single 100 mL flow-through reactor previously described by our group, where the biomass will stay immobilized for the whole ALACEN process.^40^ The reactor and system scheme is shown in Supporting Information Figure S1. Typically, approximately 16 g biomass was used if not stated otherwise. Eluent was pumped through at a constant flow to start the extraction process. For an alkaline reaction, 0.25 M NaOH (1 wt %) at 13 g_eluent_/g_biomass_, room temperature, and 6 h running time were used. A flow of 0.4 to 0.55 g/min was used, depending on the starting material. Then, 500 mL of deionized water was flown through the reactor with the biomass, and the next extraction step was initiated. The reaction with dilute acid was conducted with 11 g_eluent_/g_biomass_ (equivalent to a flow of 5 to 6 g/min; note that g_biomass_ represents the amount of biomass loaded at the start of the process before the alkali treatment) with 0.75% (w/w %) of sulfuric acid at 130 °C for 30 min and a backpressure of 5 bar. The process was carried out as follows: the eluent was run through until there was a constant flow and the backpressure reached 5 bar, the pumping of the eluent was stopped, and the electrical oven was set to start heating. Once the internal temperature of the reactor reached 130 °C (typically 7 min), the pump was started again, and that time was taken as *t* = 0. Once the desired running time was reached, the oven was cooled to 20 °C by blowing air through the oven fan, and the reactor was washed with 500 mL of deionized water. The final step of the process was the enzymatic reaction with the cellulolytic enzymatic cocktail (Cellic CTec 2) (Sigma-Aldrich). The enzymatic reaction was conducted in 500 mL of 50 mM acetate buffer at pH 5 and supplemented with 0.8 mM tetracycline chloride to avoid bacterial growth. The enzyme dosing was 3 g_protein_/100 g_biomass_, where the enzyme cocktail had a protein content of 61.2 mg_protein_/mL_enzyme cocktail_. To emulate the shaking that is normally applied, the buffer with enzyme was recirculated through the reactor for 72 h, and the liquid fraction was collected in an Erlenmeyer flask. To allow for proper enzyme dosing and mass-balancing, the biomass was removed from the reactor after dilute acid extraction. The biomass residue was dried in a vacuum oven at 60 °C until moisture was below 5 wt %. The solid was weighted and stored in a closed vial until analysis by the NREL analytical procedure.^41^ After this analysis, a measured amount of biomass was placed back into the flow-through reactor for the next step. In principle, the whole process can be run by simply changing the liquid feeds in sequence (including washings) without the need of removing the biomass from the reactor at any point.

### Chemical Composition of the Soluble and Insoluble Fractions

Insoluble fractions and initial biomass compositions were determined using the laboratory analytical procedure of the National Renewable Energy Laboratory (NREL, USA).^41^ The monosaccharides were detected by high-performance liquid chromatography (HPLC) using an Agilent 1200 pump equipped with a Bio-Rad organic acid column (Aminex HPX-87H), a refractive index detector, and a UV detector (210 nm). The HPLC column was operated at 60 °C, and a 5 mM aqueous sulfuric acid solution was used as the mobile phase, with a flow rate of 0.55 mL/min. The injection volume was set at 5 μL. The concentrations of individual compounds in the product mixture were determined by using calibration curves obtained by analyzing standard solutions of known concentrations.

FA and CA concentrations in the alkaline eluent after filtering with 0.2 nm nylon filters were analyzed by HPLC in a Hewlett-Packard 1100 Series instrument with a DAD detector at 210 nm. The column used was a ZORBAX Eclipse XDB-C18 250 × 4.6 mm 5 μm operated at 60 °C with 80%/20% water acetonitrile and 1.5 v/v % of glacial acetic acid. To quantify the initial FA and CA in the biomass, 10 mL of 2 M NaOH was added to 100 mg of biomass and mixed under a nitrogen atmosphere and darkness overnight, and the liquid was adjusted to pH 5 and introduced to the previously mentioned system.

Soluble hemicellulose sugar-rich fractions were filtered through 0.2 μm nylon filters, diluted 10 times, and analyzed using the above-mentioned Agilent 1200 HPLC system. Since this system can only quantify monosaccharides, the quantity obtained after the direct injection was considered the quantity of xylose in the monosaccharide form. To detect the total quantity of xylose in the soluble stream, the stream was hydrolyzed with 4% H_2_SO_4_ for 1 h at 120 °C. The fraction of monosaccharide xylose was measured as the difference between the total xylose and the monosaccharide xylose divided by the total xylose.

### Residual Enzymatic Lignin Isolation and Lignin Analysis

Residual enzymatic lignin (REL) was obtained as described previously by our group.^42^ Briefly, 15 g of biomass was milled for 24 h (effective milling time) in a planetary ball mill (Fritsch GmbH, Idar-Oberstein, Germany) equipped with a 250 mL ZrO_2_ jar and ZrO_2_ balls (5 × 15 mm, 10 × 5 mm). A program with 450 rpm rotation, interchanging between 10 min milling and a 15 min pause, was used. After milling, 5 g of the biomass was hydrolyzed using Cellic CTec 2 in 50 mM acetate buffer at pH 5 and supplemented with 0.8 mM of tetracycline chloride to avoid bacterial growth for 72 h. The enzyme dosing was 3 g protein/100 g biomass, and the solid-to-liquid ratio was 30. After the enzymatic reaction, the lignin was separated from the carbohydrate-rich liquid and washed four times with water by centrifugation in a Thermo Scientific megafuge 40 centrifuge with a 75003607 rotor at 450 rpm for 15 min. The final solid was dried in a vacuum oven at 50 °C overnight.

To prepare the lignin-rich solid obtained after the ALACEN process for gel-state NMR analysis, the dried biomass was milled for 30 min using the same protocol as for the REL milling; after milling, the sample was ready for NMR analysis. 2D HSQC NMR of REL and the residual lignins of the ALACEN process were collected on a 600 MHz Bruker Biospin (BASIC PROBHD, Rheinstetten, Germany) instrument. A 40 mg sample was swelled in a 0.6 mL mixture of DMSO-*d*_6_ and pyridine-d5 (4:1). Bruker standard pulse sequence ‘hsqcetgpsisp.2′ was used for the ^13^C–^1^H correlation experiment. Reported parameters with minor modifications were used for the analysis: spectra use 2048 data points from 11 to 0 ppm in F2 (^1^H) (acquisition time 130 ms) and 160 to 0 ppm in F1 (^13^C) with 256 increments (acquisition time 6 ms) of 32 scans with 500 ms internal delay; the d1 delay was set to 86 ms. The total acquiring time is 3.54 h.^42^ The signal of DMSO-*d*_6_ was used as an internal reference (F2 δ 39.5 ppm, F1 δ 2.49 ppm). The data were processed by MestReNova x64–12.0.4–22023.

### Catalytic Hydrogenolysis

The process was conducted as previously reported.^43^ In brief, 50 mg of lignin was added to the custom-made 10 mL reactor together with 4 mL of absolute methanol, 25 mg of 5 wt % Ru/C catalyst, and a magnetic stirring bar. Then, 60 bar of hydrogen was loaded, and the reactor was heated at 250 °C and stirred at 300 rpm in an oil bath for 3 h. The reactor was cooled, and 4 mL of a 4 mM octadecane in absolute methanol was added as an internal standard. The sample was filtered using 0.45 μm polytetrafluoroethylene membrane and analyzed by gas chromatography–mass spectrometry (GC–MS) and GC-flame ionization detection (FID) under the conditions described earlier.^43^ The GC-FID chromatogram and mass spectra are shown in Supporting Information Figures S2 and S3, respectively.

### Bacterial PHA Production

*S. thermodepolymerans* DSM 15344 was purchased from the DSMZ-German Collection of Microorganisms and Cultures (Braunschweig, Germany).

The inoculum medium consisted of peptone (10 g/L), yeast extract (5 g/L), and NaCl (10 g/L).

The basic culture medium for PHA production contained (NH_4_)_2_SO_4_ (1.1 g/L) (if not stated otherwise), MgSO_4_·7H_2_O (0.45 g/L), KH_2_PO_4_ (1.31 g/L), Na_2_HPO_4_·2H_2_O (1.68 g/L), and 1.5 mL/L of Santer’s trace element solution.^44^ The initial pH value of all culture media was adjusted to 7 using NaOH. The inoculum and basic culture medium were cultured at 150 rpm and 50 °C in Erlenmeyer flasks. The basic medium was inoculated with 10% (v/v) volume for further experiments. Bacterial growth was monitored via optical density at 600 nm every 24 h, and substrate consumption was analyzed as mentioned in the previous section via HPLC to detect monosaccharide sugars. Only for the samples generated by direct dilute acid (DDA) treatment was a post-hydrolysis conducted for monosaccharide quantification via HPLC (as described in Section 2.4). For the fraction obtained by the ALACEN process, the dilute acid hydrolysate was neutralized to pH 7 and added directly to the media as the sole carbon source without further treatment.

### PHA Measurement, Extraction, and Identification

The polyhydroxybutyrate (PHB) content in the lyophilized cells was determined following methanolysis based on a previously reported protocol.^45^ The obtained methyl esters were analyzed by GC–MS on a Hewlett-Packard 6890 GC, equipped with a Rxi-5Si capillary column (30 m × 0.25 mm inner diameter and 0.25 μm film thickness). Helium was used as the carrier gas with a flow rate of 2 mL/min. Benzoic acid was used as an internal standard, and commercial PHB (Sigma-Aldrich) was used as an external standard. For the identification of the polymer produced, the cells after the last day of growth were collected via centrifugation at 12 000 rpm (Thermo Fisher, F15-6x 100y rotor) for 10 min and lyophilized. Then, the PHB was extracted using hot chloroform with a 1 to 20 (w/v) ratio at 60 °C for 48 h. The suspension was filtered over a Whatman GF/A glass filter to remove cell debris. Nine volumes of ice-cold absolute methanol were added to the filtered sample forming a white precipitate that was collected by centrifugation at 1000 × *g* for 10 min^46^ The extracted polymer was further identified by NMR (Agilent 400 MHz MR-DD2, Agilent, Santa Clara, CA, USA) with deuterated chloroform (CDCl_3_) as a solvent. ^1^H NMR spectra were recorded using 8–32 scans, 45° duration of the pulse, and 1 s repetition delay. ^13^C NMR spectra were recorded using 128 scans, 45° duration of the pulse, and 1 s repetition delay. The extracted samples were compared to commercial PHB (Sigma-Aldrich).

## Results

### Mild Alkaline Treatment

Wheat straw was selected as a model of herbaceous biomass due to its high production in Europe, where around 46% of the produced cereal crops is wheat (2013).^47^ The composition of the wheat straw used in this work was determined to be 34% glucan, 27% hemicellulose, 17% lignin, 0.5% CA, and 0.3% FA based on dry weight (see Supporting Information Figure S4). This composition fits the data reported for typical herbaceous biomass (see Supporting Information Table S1).^48^

The use of alkaline pretreatment has been widely studied in the literature.^62^ Such pretreatment can increase glucan digestibility using high NaOH concentrations (15–20%) and high temperatures (145–170 °C),^30,63,64^ however, these lead to extensive degradation of lignin and hemicellulose-derived sugars.^65^ By using mild (room temperature) diluted (1–2.5% NaOH) conditions, it is aimed to specifically remove the esterified compounds with two objectives: (1) obtain a highly valuable stream enriched in these hydroxycinnamic acids together with acetic acid,^66^ (2) weakening of the interlinkages between hemicellulose and the other components as well as inducing physical changes that facilitate the subsequent fractionation steps.^10,67^ Both effects are tested here in a flow-through setup described in the Supporting Information (Figure S1).

**Figure 3:**
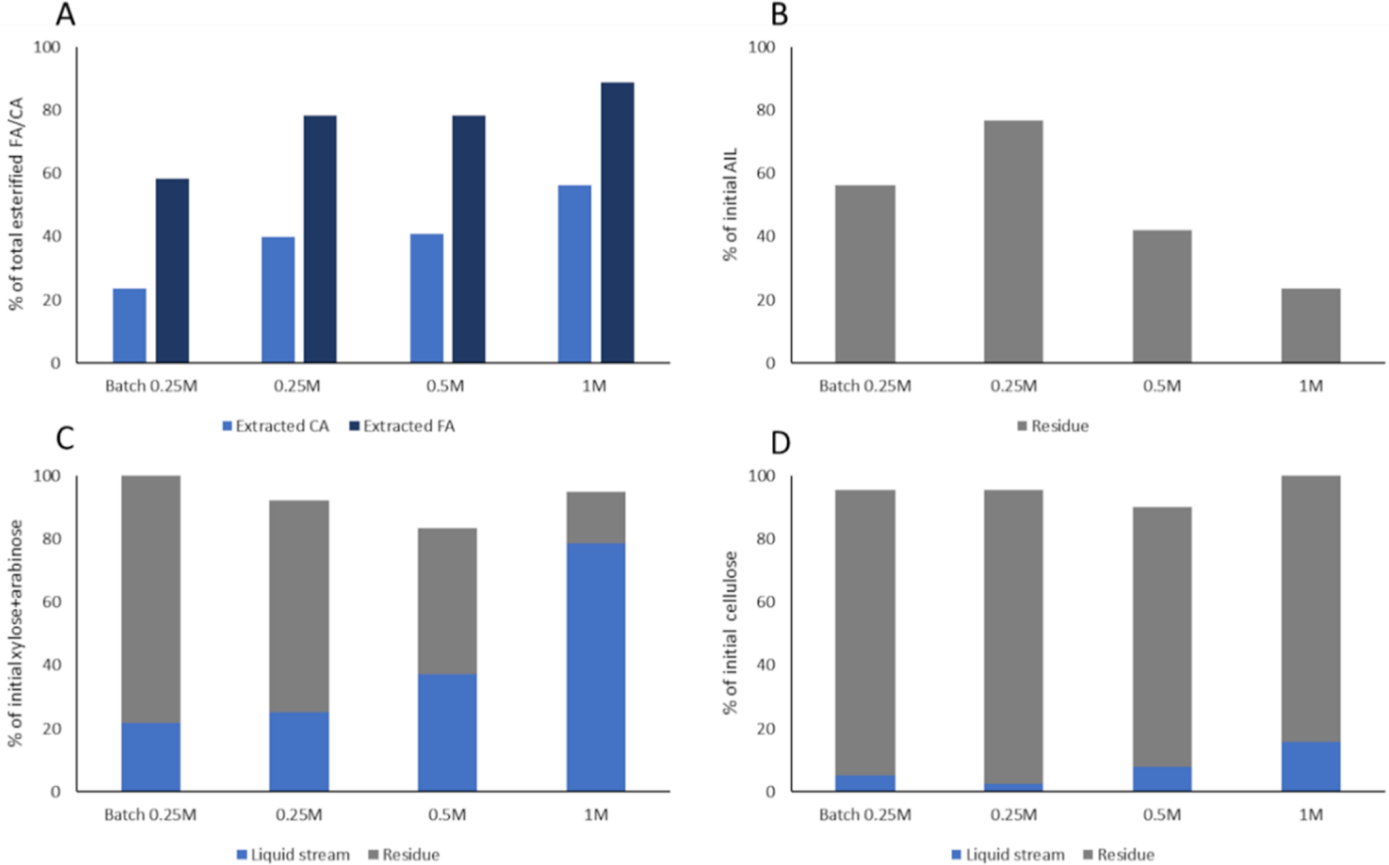
Effect of different alkaline concentrations at room temperature for 6 h on wheat straw. The residue compositions are measured by NREL protocol, while the carbohydrates in the liquid were measured in HPLC after post-hydrolysis of the super natant. (A) Yield of esterified FA and CA solubilized as free FA and CA. (B) Amount of acid-insoluble lignin present in the residue after alkaline treatment. (C) Amount of xylose and arabinose remaining in the pellet after treatment (residue) and present in the alkaline stream (liquid stream). (D) Amount of glucose remaining in the pellet after treatment (residue) and present in the alkaline stream (liquid stream).

The effect of the mild diluted alkaline in flow-through was compared to a conventional batch system with 0.25 M NaOH (Figure 3A). In the flow-through system, 78% FA and 40% CA removal was achieved with 23% and 24.7% loss of lignin and xylan, respectively (Figure 3B,C). When the reaction with the same conditions was conducted in a batch reactor, 44% of the lignin was solubilized, obtaining only 58% of FA and 23% of CA removal (Figure 3A,B). The flow-through extraction with 0.25 M NaOH presents a greater capacity to obtain high FA removal with relatively good retention of the other biomass components. The increased selectivity of the flow-through system can be seen not only compared to our own data but also to kinetic models in batch systems. Those predict for rice straw (0.2 M NaOH, 30 °C and 6 h) that to obtain an 80% FA removal yield, 45% of the lignin and 35% of the hemicellulose will be solubilized.^49^ Thus, the results highlight the benefit of the flow-through system over the batch system for the selective removal of esterified compounds.

An increase in the NaOH concentration did not produce a significant rise in the removal yields, especially between 0.25 and 0.5 M, there was only an increase of 0.6% and 0.2% for CA and FA, respectively. A similar low effect in the FA and CA release by increasing alkaline concentrations was previously reported in rice straw^49^ and corn bran.^50^ A further increase in the NaOH concentration to 1 M did lead to higher FA and CA removal (88.8% and 52.2%) at the expense of other biomass components (Figure 3B–D). Lignin was the most affected component, with 0.25 M NaOH 77% of the initial acid-insoluble lignin remained, while with 1 M only 24% remained (Figure 3B). In particular, the acid soluble lignin (ASL) as determined by the NREL protocol was more readily removed. Only 32% of the ASL remained after the 0.25 M treatment and 17% in the 1 M treatment (Supporting Information Table S1). The difference between the ASL and the acid insoluble lignin (AIL) removal in the alkaline step is likely because FA and CA have a maximum UV absorbance at 320 nm, the same wavelength used for ASL quantification according to the NREL protocol.^41^ Therefore, the alkaline extracted hydroxycinnamic acids are likely a major contributor to the ASL in the initial biomass, and we opted to focus on using only the AIL retention.

Increasing the NaOH concentrations also results in the undesired higher solubilization of hemicellulose components, with 0.25 M, only 24.7% of the initial hemicellulose-derived sugars were dissolved, while for 1 M, 77% was solubilized (Figure 3C). Finally, as known for these types of alkaline pretreatments, the glucan fraction remained nearly unaffected for all the alkaline concentrations^51^ (Figure 3D). Due to the high solubilization of FA and CA (78 and 40%) and the retention of 77% AIL and 76% hemicellulose components (Supporting Information Figure S5), the biomass treatment at 0.25 M and room temperature for 6 h in a flow-through system was chosen as the best conditions for the first fractionation step.

### Mild Dilute Acid Treatment

**Figure 4:**
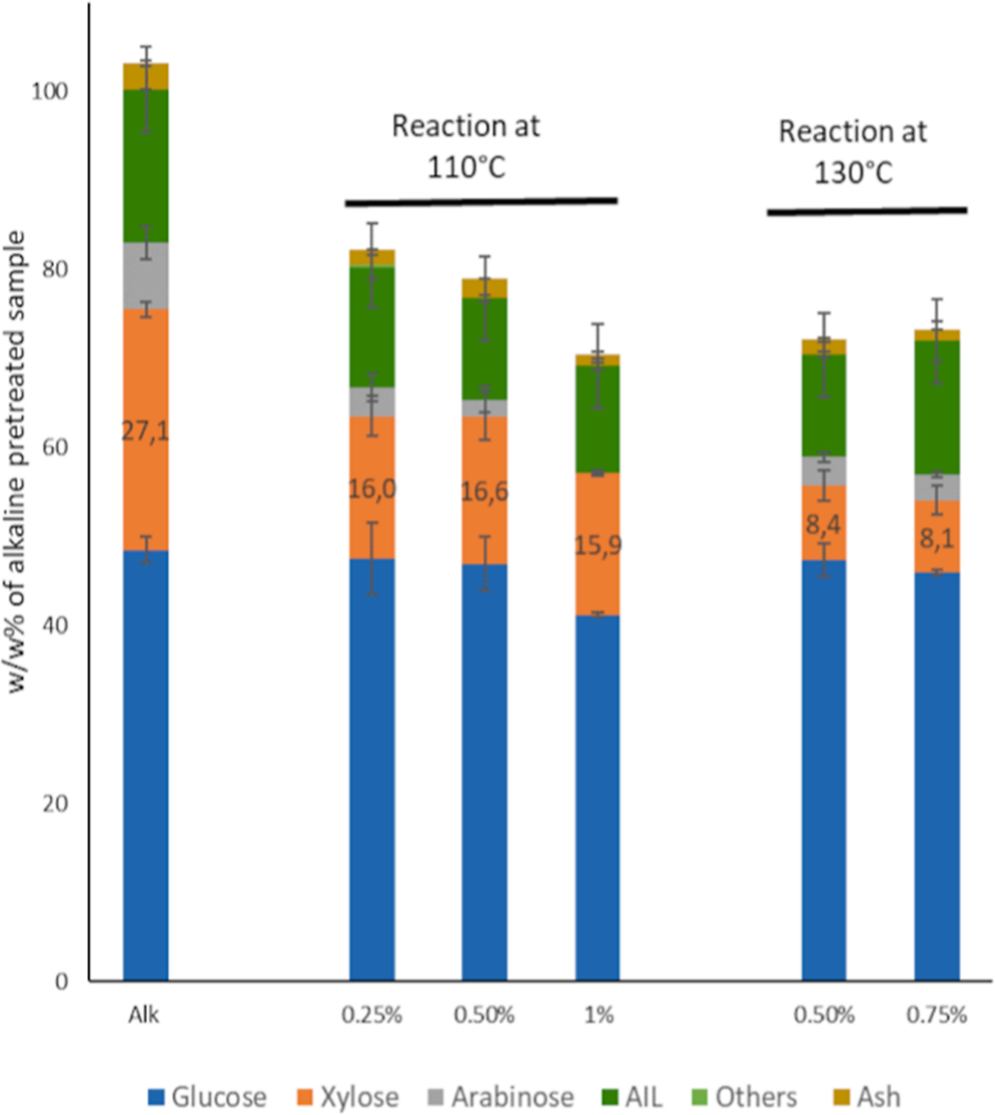
Effect of dilute acid treatment on 0.25 M alkaline-pretreated wheat straw (Alk). For all the treatments, the time was 30 min with 15 g_eluent_/g_alkaline-pretreated biomass_ loading at different H_2_SO_4_ concentrations (0.25–1 w/w %) and different temperatures of 110 and 130 °C. The w/w % of each component after the treatment was compared to the initial weight of the alkaline-pretreated sample. The standard deviations of 0.5, 0.75% at 130 °C as well as the alkaline reaction are from 3 or more independent experiments; the others represent the analytical error of the NREL of the residue obtained from a single experiment.

Dilute acid hydrolysis is a well-developed process to solubilize the hemicellulose fraction with a minor impact on lignin and glucan.^52^ In most of the literature, temperatures between 140 and 160 °C and 1–2 w/w % of H_2_SO_4_ are reported.^30,32,53,54^ These harsh conditions usually generate fermentation inhibitors such as hydroxymethylfurfural (5-HMF), furfural, and formic acid.^32,33^ Here, we opted to test dilute acid treatment with low acid concentration (0.25–0.75 w/w %) and low temperatures (110–130 °C) to completely avoid the formation of the sugar degradation products (Figure 4).

The hemicellulose solubilization was nearly 70% at 130 °C with 0.75% H_2_SO_4_. A lower acid concentration of 0.5% decreased hemicellulose solubilization by only 0.6%. A milder temperature of 110 °C gave a significantly lower hemicellulose solubilization of 38–43%. The highest hemicellulose solubilization at 110 °C was 43% when 1% H_2_SO_4_ was used but this led to the undesired loss of 15% of the initial glucose.

If the wheat straw was subjected to DDA with 0.75% H_2_SO_4_ at 130 °C for 30 min, the solubilization of hemicellulose-derived carbohydrates was significantly lower (57% compared to 70%; see Supporting Information Figure S4), showing an increase in the performance of the diluted acid after alkali treatment. However, if accounting for the losses in the alkali treatment, the ALACEN process has a similar efficiency of recovery of hemicellulose-derived carbohydrates (55%) compared to the DDA treatment (although the composition of this stream is very different, vide infra). These results are also corroborated by previous reports in batch, wherein the pretreatment of wheat straw with similar mild conditions yielded 45% of hemicellulose solubilization compared to the 70% obtained in the ALACEN process^32^ with wheat straw.

The lignin and glucan components were only reduced by 14 and 6%, respectively, with 0.75% H_2_SO_4_ at 130 °C. Moreover, no dehydration products like furfural and 5-HMF were detected (below the 10 μg/mL detection limit) for all of the conditions tested. The use of a continuous flow-through setup in combination with the mild conditions thus showed good selectivity toward solubilizing hemicellulose avoiding the secondary reactions.

**Figure 5:**
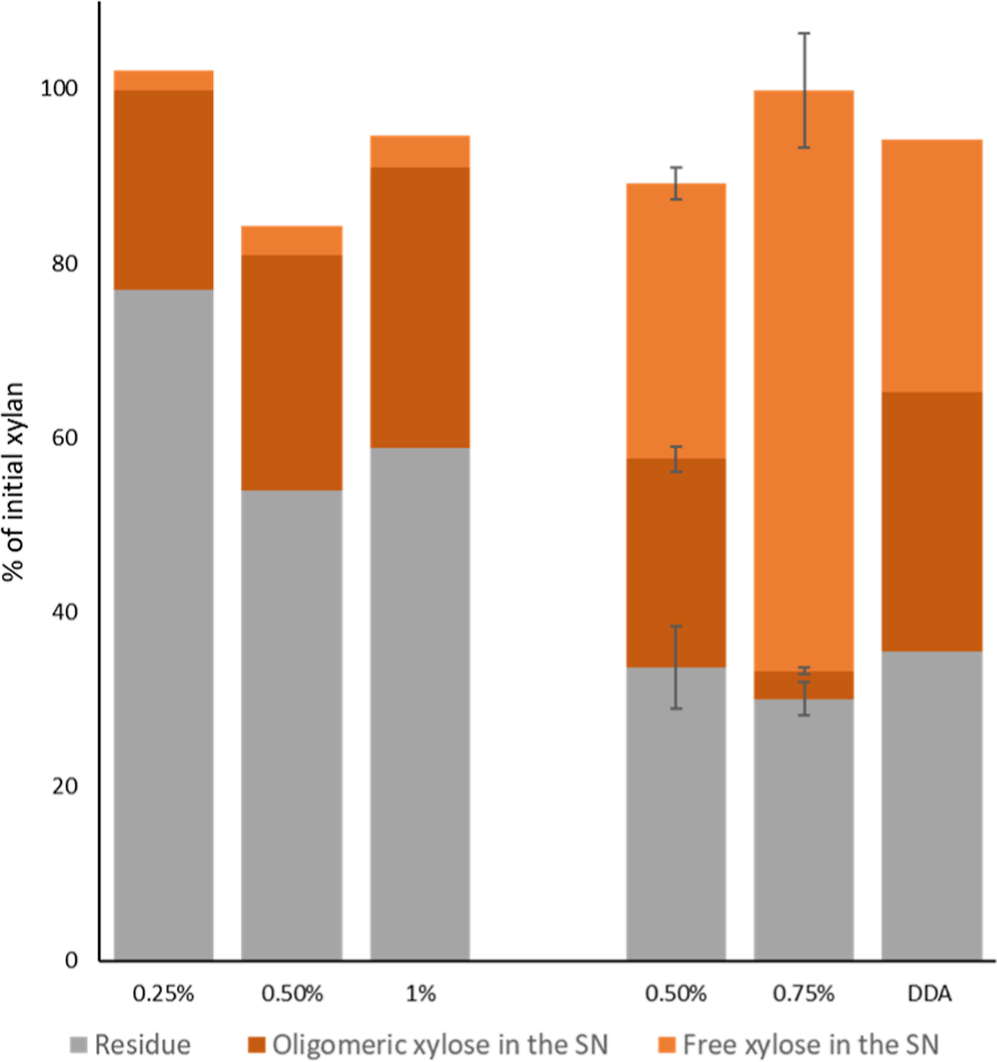
Xylose distribution of dilute acid treatment for 0.25 M alkaline-pretreated wheat straw at 110 °C (first three columns) and 130 °C (second three columns) with different sulfuric acid concentrations (w/w %). Free xylose represents the xylose detected by HPLC after direct injection of the dilute acid supernatant. Oligosaccharide xylose is calculated by the difference between the total xylose present in the supernatant, detected by HPLC after post-hydrolysis minus the quantity of free xylose determined by HPLC without post-hydrolysis. Residue samples are calculated following the NREL protocol. For the DDA, the conditions were the same as those for the dilute acid with 0.75% H_2_SO_4_ but without previous alkaline pretreatment. Standard deviations are shown only for treatments with 3 or more independent batches.

At 0.75% H_2_SO_4_ and 130 °C, the conversion of the alkali-pretreated biomass achieved a stream containing 95% of the released carbohydrates as monomers, obtaining 51% of the total initial xylose as free monomeric xylose (Figure 5). In contrast, reactions at 110 °C gave little free monomeric xylose, and the DDA yielded only 49% monomeric xylose, which corresponds with the expected value for the conditions used.^54,55^ The direct production of monosaccharides with no furanics and cinnamates is of significance as this avoids the requirement of a post-hydrolysis step to convert oligosaccharides into monosaccharides in addition to avoiding a purification step to remove inhibitors.^56^ Due to the balance between hemicellulose solubilization as free monosaccharides, conservation of the rest of the biomass compounds, and the lack of formation of potential fermentable inhibitors, the combination of mild diluted alkaline (0.25 M NaOH at room temperature for 6 h) and dilute acid reaction with 0.75 w/w % sulfuric acid at 130 °C was selected as the optimal set of conditions for the ALACEN fractionation process (Supporting Information Figure S5).

### Conversion of the Crude Monomeric Xylose Stream to PHA

To showcase the valorization potential of the xylose-rich hydrolysate from the ALACEN process, the obtained crude neutralized xylose-rich stream was used as the sole carbon source for the production of PHA by the thermophilic bacterium *S. thermodepolymerans*. Xylose-utilizing bacteria are not common and even less common with industrially relevant capacities like *S. thermodepolymerans,* which is able to produce PHA at 50 °C with xylose as the main carbon source.^15,57^ The PHA production was compared to that of using pure xylose as the sole carbon source.

**Figure 6:**
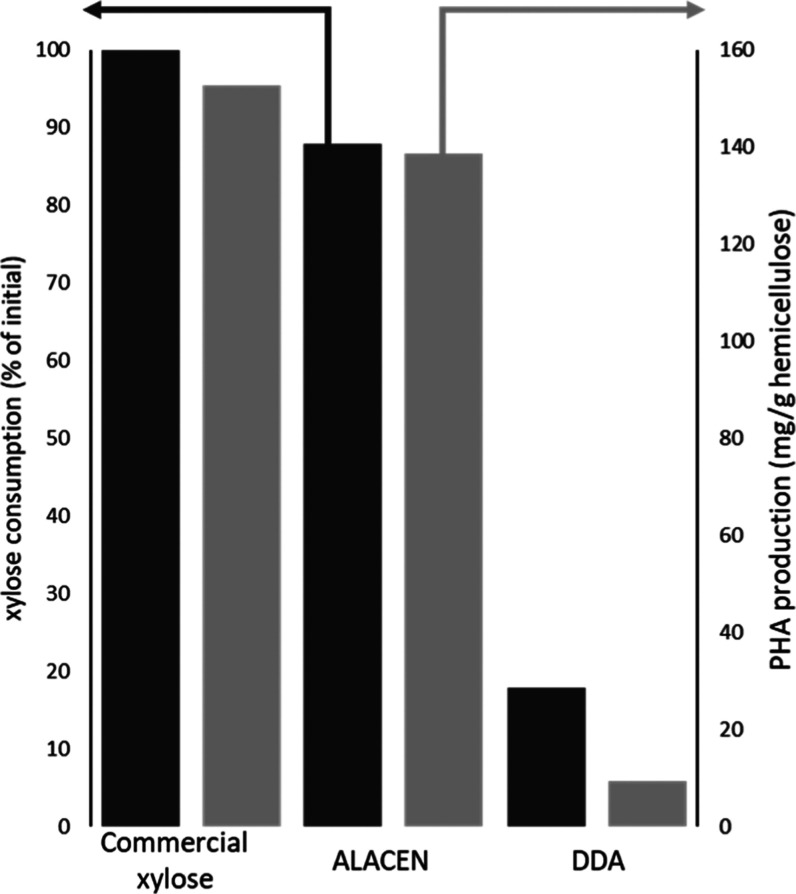
PHA production (gray bars) and xylose consumption (black bars) with *S. thermodepolymerans* at 50 °C using mineral media supplemented with different carbon sources: 5 g/L of commercial xylose, ALACEN: dilute acid stream produced with the optimum conditions of the ALACEN process on wheat straw, DDA is the stream originated from the DDA treatment of wheat straw in the flow system at 130 °C for 30 min with 0.75% H_2_SO_4._ Hemicellulose accounts for the quantity of xylose + arabinose. All PHB production experiments were carried out in duplicates and showed an error lower than 7%.

The crude dilute stream obtained in the ALACEN fractionation containing 9.7 ± 0.4 g/L of xylose and 2.1 ± 0.1 g/L of arabinose was used as the only carbon source for PAH production after neutralization and minimum mineral media salt addition. As shown in Figure 6, *S. thermodepolymerans* consumed 88% of the xylose and 66% of the arabinose after 96 h of incubation., this value is close to the nearly 100% obtained with commercial pure xylose. Not only was the xylose stream consumed but it also matched 90% of the production of PHA per gram of carbohydrate input (xylose and arabinose) compared to pure commercial xylose, confirming the absence of fermentation inhibitors (Figure 6). NMR analysis combined with analysis by GC–MS after methanolysis of the extracted material confirmed that the main component of the recovered PHA was indeed PHB (Supporting Information Figure S6).

The properties of the obtained PHA have been studied and compared with those of commercial xylose, which has been reported by Zhou et al. (2023).^15^ Both of the produced PHA from xylose-rich stream and commercial xylose are identified as poly[3-hydroxybutyrate (PHB)] by NMR (Supporting Information Figure S6). The analysis performed by thermogravimetric analysis (TGA) (Supporting Information Figure S7) and differential scanning calorimetry (DSC) (Supporting Information Figure S8) shows that the glass transition temperature (*T*_g_) and melting temperature (*T*_m_) of the obtained PHA are −29 and 166 °C, respectively. The properties are similar to the commercial PHA measured by the group^15^ (*T*_g_ at −29 °C and *T*_m_ at 170 °C).

To investigate the beneficial effect of the full ALACEN process, including the initial mild diluted alkaline treatment, PHA production was performed with the stream obtained from DDA treatment, so without preremoval of esterified hydroxycinnamates. When alkaline pretreatment was not performed, only 18% of the initial hemicellulose derived carbohydrates were consumed, leading to a poor PHA production of 9.4 mg_PHA_/g_hemicellulose_ in comparison with 139 mg_PHA_/g_hemicellulose_ in the double-treated wheat straw (Figure 6 and Supporting Information Figure S9). This can be attributed to the DDA hydrolysate containing 50% of the total xylose in oligosaccharide form, in comparison with the 5% in the fraction obtained with the ALACEN process (Figure 5, Supporting Information Figure S9). Another reason is likely the lack of removal of FA in the DDA sample, which is known as a strong growth inhibitor for *S. thermodepolymerans*.^12^

Overall, ALACEN represents an interesting opportunity to produce a simple crude xylose-rich stream suitable for PHA production with thermophilic bacteria (Supporting Information Figure S5).

### Enzymatic Saccharification of Glucan and Analysis of the Native-like Lignin Residue

**Figure 7:**
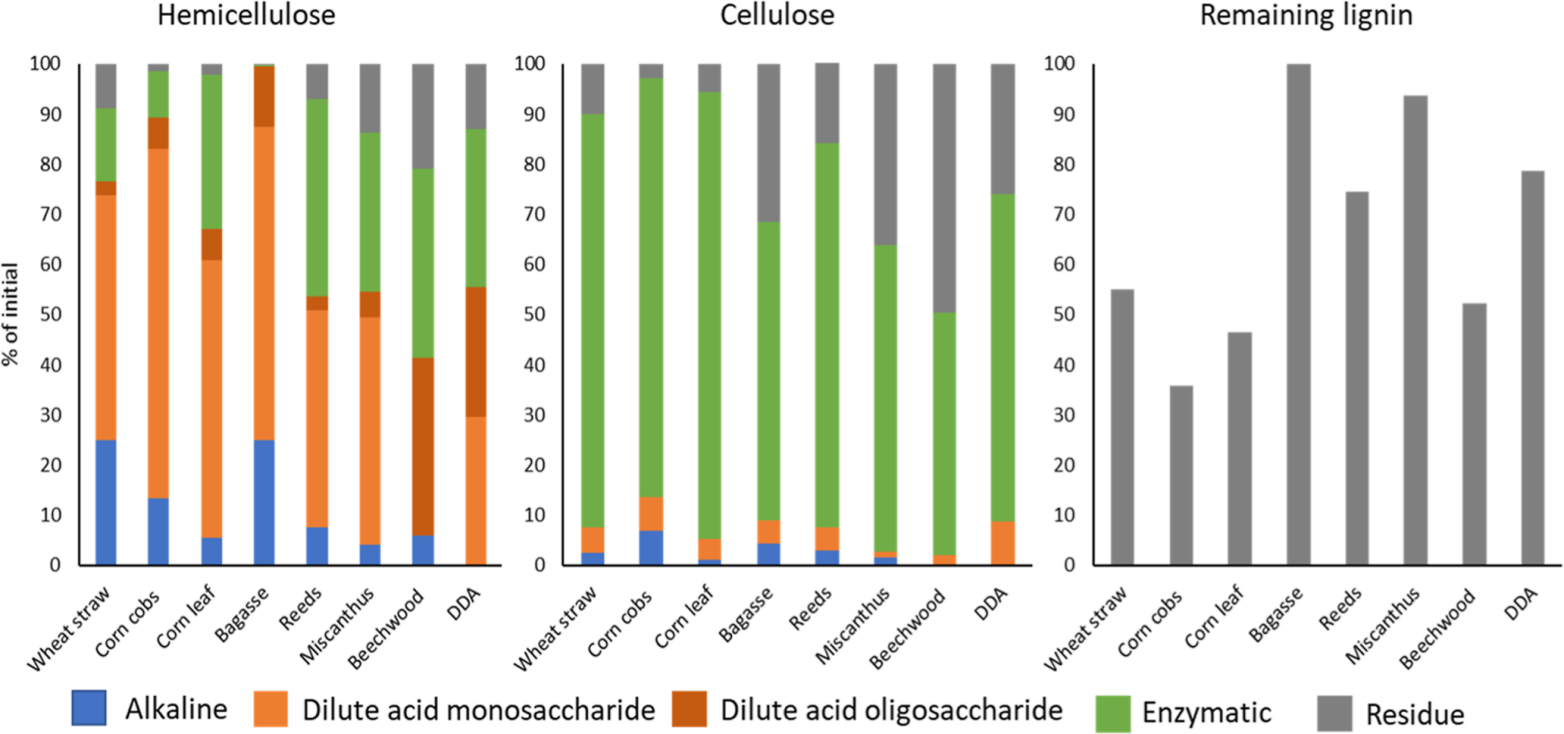
Distribution of xylose + arabinose, glucose, and acid-insoluble lignin for the full fractionation process on different biomasses. All of the biomasses were treated in a flow reactor with 0.25 M NaOH at room temperature for 6 h (alkaline). The residue was treated at 130 °C with 0.75 w/w % of H2SO4 for 30 min (dilute acid), and the remaining residue was hydrolyzed with CTec 2 enzymatic cocktail for 72 h with a 3.0 w/w % of enzyme to biomass loading (enzymatic hydrolysis of glucan), leaving the last residue in the reactor (residue). To avoid the interference of the carbohydrates contained in the enzymatic cocktail, the carbohydrates released from the enzymatic treatment are expressed as the difference between the carbohydrate content measured in the starting material (after alkali and dilute acid treatment) and the residue as determined by NREL. The quantity of each component after each treatment is found in Supporting Information Table S1.

The final step of the ALACEN process was to evaluate the effect of the pretreatments on the value potential of the residual lignin-cellulose residue. Enzymatic saccharification of the glucan retained in the residue after the alkali and acid treatments was conducted in the same flow-through system. The output stream of the system was connected to the eluent reservoir to create a recirculation mode. After 72 h of enzymatic hydrolysis with Cellic CTec 2, 82% of the initial glucan in wheat straw was obtained as glucose, with an enzymatic reaction efficiency of above 90%. When compared to the saccharification of wheat straw DDA residue, the introduction of the dilute alkaline treatment represented an increase of 17% in the yield of glucose obtained from the initial biomass (Figure 7). This indicates that the alkali treatment has an additional beneficial effect on enzymatic saccharification. This could be due to the removal of esterified compounds, as has been previously observed in the literature.^10^

**Figure 8:**
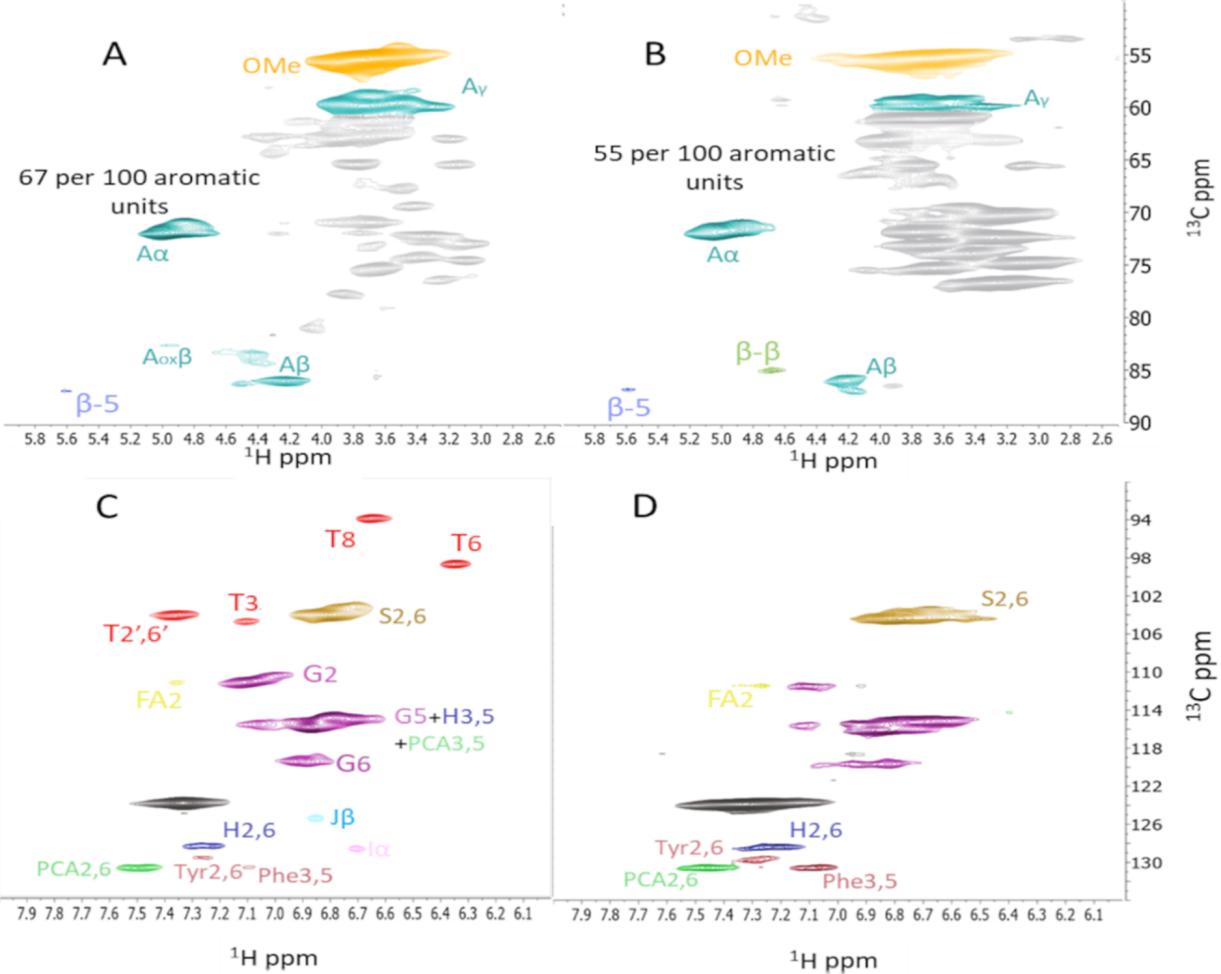
Gel state HSQC of the REL (A,C) and the lignin obtained after the ALACEN process from wheat straw (B,D). The main linkages and groups detected are A: β-O-4 alkyl-aryl ethers, Aox: Cα-oxidized β-O-4, β-5:phenylcoumarans, β–β:resinols, T: tricin, Phe: phenylalanine, Tyr: tyrosine, FA: ferulic acid, pCA: p-coumarate, I: cinnamyl alcohol end-groups, and J: cinnamyl aldehyde end-groups. The peak assignment and structure were performed according to.^44^

The high glucose production in ALACEN is at the same level as that obtained by the dilute acid-alkaline process^29,59^ and produces more glucose than 30–65% using single pretreatments.^53,64^ Interestingly, the enzymatic saccharification of the lignin-cellulose residue showed such high efficiency, while 55% of the initial lignin remained in the pellet (Figure 7). It is known that lignin has an inhibitory effect on glucose degradation.^82,83^ Recently, however, it was shown that noncondensed lignins had no such inhibitory effect.^42,84,85^ Under optimized conditions in wheat straw, the ALACEN process still retains 55% of the initial lignin in the residue. This lignin has a high β-O-4 conservation of 55% β-O-4 motifs per 100 aromatic units. This represents a loss of only 11.7 β-O-4 motifs per 100 aromatic units in comparison with the native-like REL lignin obtained from the same biomass (Table 2, Figure 8). Thus, the high conservation of lignin in the ALACEN process allows for high cellulose saccharification in the presence of high quantities of lignin.

**Table 1:** Concentration of FA, CA, and Acetic Acid Obtained in the Alkaline Stream of the ALACEN Process for Different Biomasses^a^

biomass	FA mg/g biomass^b^	CA mg/g biomass^b^	acetic acid mg/g biomass
wheat straw	1.8 (78.1%)	1.4 (40.1%)	27.2
corn cobs	8.3 (78.2%)	17.0 (61.8%)	40.0
bagasse	2.1 (89.0%)	8.6 (62.1%)	30.0
reeds	1.9 (55.1%)	5.4 (33.5%)	24.6
miscanthus	2.0 (nd)	6.6 (nd)	19.2
corn leaf	4.5 (nd)	4.5 (nd)	20.9
beechwood	0.0 (0.0)	0.0 (0.0)	37.7
direct wheat	0.3(8.4%)	0.3 (5.7%)	18.6
corn fiber	12.6 (52.9%)	1.5 (44.2%)	nd

a Between parentheses is found the yield of FA/CA from the initial quantity in the biomass.

b Measured only as free FA and free CA, % values indicate the percentage of initial FA and CA. nd: not determined.

**Table 2:** Semiquantitative Analysis of the Lignin Obtained with the ALACEN Process on Different Biomasses Using Gel-State HSQC NMR with DMSO/Pyridine (4:1)^a^

biomass	S	G	H	β-O-4^d^	β-5^d^	β–β^d^
REL^b^	44	51	4	67	5	8
wheat straw	60	37	3	55	7	8
corn cobs	62	36	2	32	0	4
bagasse	51	47	2	43	5	9
miscanthus	43	55	3	49	4	13
corn leaf	79	21	1	18	6	5
DDA^c^	49	49	2	60	4	0

a All the raw spectra can be seen in Supporting Information Figures S12–S18.

b Residual enzymatic lignin.

c Direct dilute acid.

d Linkages are represented as number of linkages per 100 aromatic units.

These levels of lignin structural preservation are higher when compared to the 7.8–31% usually obtained with alkaline pretreatments^86,87^ or similar to the 56% obtained with γ-valerolactone extractions,^88^ but still a little under the best mild orgnasolv conditions.^40,89,90^ The high β-O-4 content in the recovered lignin suggests that this lignin is suitable for depolymerization of valuable aromatic monomers via selective depolymerization processes like catalytic hydrogenolysis.^58^ When applying the conditions of this reported procedure (Ru/C, 250 °C, 60 bar H_2_, and 3 h), similar yields of selective aromatic monomers are obtained for ALACEN and REL lignins (14 and 13%, respectively, see Supporting Information Figure S11). This shows additional evidence of the mild impact of the biomass fractionation process described herein has on the native structure of lignin. Another factor to take into consideration is the ease of lignin recovery in the ALACEN process. Here, the lignin is retained in the reactor in the solid state, avoiding the time-consuming and tedious precipitation and filtration of the lignin.

### Application of ALACEN to Other Biomasses

Next, the ALACEN fractionation process was extended to different herbaceous lignocellulosic biomass residues (corn cobs, corn leaf, miscanthus, sugar cane bagasse, and reeds) to explore its broader applicability. Additionally, a nonherbaceous biomass (beechwood) was used for comparison. All of these biomasses have different lignocellulose compositions that could affect the performance of the process and likely require further fine-tuning. For the sake of simplicity, the optimal conditions established for wheat straw were applied.

The yield of FA obtained by the ALACEN process (Figure 3, Table 1) is in the high range compared to the yields observed in the literature with alkaline, enzymatic, and deep eutectic treatments in several biomasses, where the ranges are between 0.3 and 13.32 mg_FA_/g_biomass_^49,50,68−71^ (only monomeric FA and CAs were quantified). As an example, Pazo-Cepeda et al.^72^ obtained a maximum yield of 8.8% from wheat bran using a pressurized aqueous ethanol solution. Ferri et al.^70^ used an enzymatic approach on wheat bran, obtaining 57% of the FA per gram of biomass obtained in this study, while Gopalan and Nampoothiri^73^ used an enzymatic biorefinery process for wheat bran, obtaining a maximum of 35% of the initial FA.^74^ FA is a product of great interest for biomass valorization due to its high economical value. Unfortunately, low concentrations are obtained due to the limitations invoked by the low initial quantities present in several agricultural residues. As an example, when using a FA-rich biomass like corn brand, the ALACEN process can obtain up to 12 mg_FA_/g_biomass_ or 8.3 mg_FA_/g_biomass_ with corn cobs, showing the good performance of this process.

Even though the quantities and concentrations obtained are quite low to develop a feasible economic process,^75^ the development of selective FA and CA removal is of great interest. One of the main limitations in the purification of FA from alkaline streams is the viscosity given to the solution by the hemicellulose cosolubilized with the hydroxyminic acids.^50,75,76^

The remaining hemicellulose fraction was largely solubilized in the subsequent dilute acid step of the ALACEN process. Between 45 and 75% of the initial hemicellulose was solubilized, and above 85% of the solubilized xylan was found as xylose for all the herbaceous biomasses (Figure 7). Yields of around 60% xylose were observed for corn-derived biomass streams (corn leaf and corn cobs) as well as for bagasse. The yields decreased to around 45% in reeds and miscanthus and were lowest in beechwood, where only 35% of the initial xylan was solubilized and mostly in oligosaccharide form. These results show that dilute acid conditions will need to be optimized for each feedstock to maximize hemicellulose solubilization and a crude xylose monomer-rich stream. In general, to achieve a yield of monomeric xylose similar to that obtained in the ALACEN process with all the biomasses (Figure 7), either higher temperatures, higher acid concentration, or longer residence times are used.^77,78^

Even though the low production of furfural at 130–140 °C with mild dilute acid conditions has been previously reported,^32^ the combination of these mild conditions with the use of a flow-through reactor in the ALACEN process brought the concentration of furfural under the detectable limit for all our streams. Flow-through systems are known for their capacity to minimize side reactions due to the reduced exposure of the solubilized compounds to the reaction temperature.^79,80^ The high quantities of monomeric xylose and the low presence of potential inhibitors like furfural,^36,81^ FA, and CA^12,37^ make the xylose stream obtained in the ALACEN process a good candidate for direct bacterial conversion to value-added products. One of the main advantages of this is that there is no need to do any hydrolysate purification or concentration.^20,56^

The advantages of mild alkaline biomass conditioning combined with a dilute acid treatment have already been proposed.^91,92^ Another advantage of these combined pretreatments is the reduction of chemicals needed for the fractionation. In specific, the ALACEN process uses 23.6% of the acid and 43.5% of the alkaline in the current xylose commercial process (Supporting Information Table S2).^20^ Even though the ALACEN process is operated at laboratory scale, the process does show promise when looking at the green metrics (Supporting Information Note 1). However, several steps still need to be further optimized. One of which is the use of water, which is higher in comparison with industrial processes, or an alternative to dewaxing and milling steps, which were not tested here.^91^

**Figure 9:**
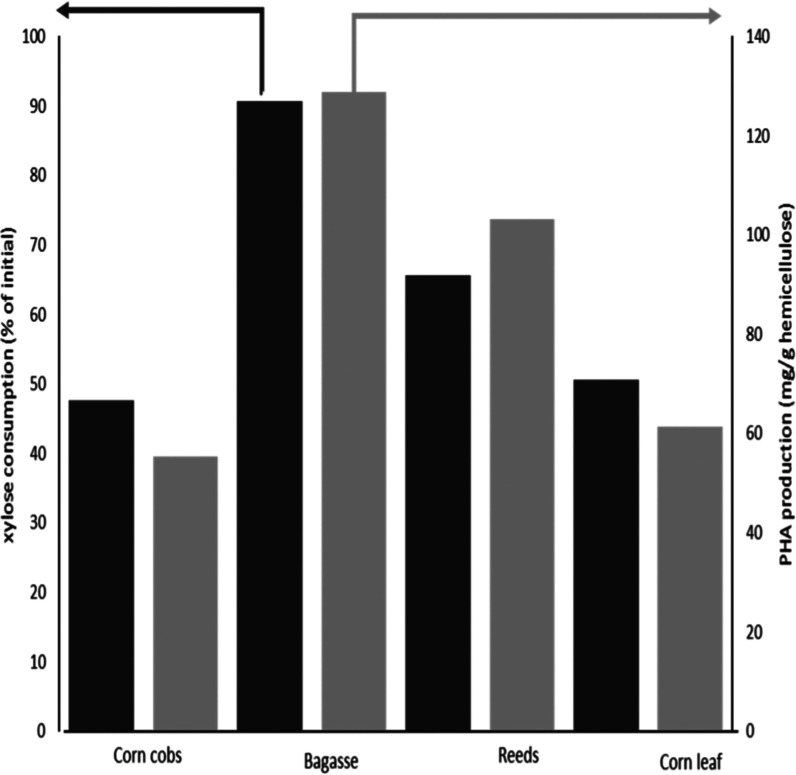
PHA production with *S. thermodepolymerans* at 50 °C using mineral media supplemented with a neutralized dilute acid stream as carbon sources coming from the ALACEN process in different biomasses. Hemicellulose accounts for the quantity of xylose + arabinose. Corn cobs, sugar cane bagasse, reeds, and corn leaf. PHA production was carried out in duplicates using one set of experiments as substrates; the error was under 5%.

The milder strategies used in the ALACEN process allowed for the direct application of the xylose-rich stream for PHA in most of the biomasses tested (Figure 9). Especially, bagasse and reeds show great potential for the application of the ALACEN process for the production of PHA, conserving between 92 and 75% of the activity obtained with the streams from the optimized wheat straw. The utilization of xylose-rich hydrolysate to PHA has been of high interest in recent years,^93−98^ but due to the difficulty of most bacteria in metabolizing xylose, there is still a lot of room to improve.^13^ Here, we show the utilization of a recently described xylose-preferring thermophilic bacteria under optimal PHA production conditions.^15^ The combination of the utilization of the xylose stream with the new ALACEN process can be of high interest to further develop better holistic valorization of biomass strategies.^13^ As we have shown, the properties and yield of PHB production are similar when directly using the xylose-rich stream and commercially purified xylose. To our knowledge, this is one of the first examples of the direct utilization of a dilute acid hydrolysate to produce PHA that shows such low inhibitory effects in comparison with commercial xylose.^8,99^ The low inhibition effect of the ALACEN hydrolysate is due to two main factors that are a direct result of the presented approach. On one hand, there are below-detectable limits for carbohydrate degradation products like furfural due to the combination of mild conditions and a flow-through system. On the other hand, the removal of phenolic compounds like FA and CA can help with bacterial growth; in specific, the bacteria used in this study, *S. thermodepolymerans*, is strongly inhibited by FA.^12^ This is one of the main reasons we think the PHA production behaves differently in the different biomasses. In specific, PHB production is lower in corn cobs and corn leaf, which have higher initial quantities of FA and CA (0.3% in wheat straw vs 1% in corn cobs).

The enzymatic saccharification of glucan in the lignin-cellulose residue was tested (Figure 7). In particular, corn cobs, corn leaf, and wheat straw hydrolysis showed an above-80% glucose yield from the initial glucan in the biomass, which entails a glucose yield of around 90% from the alkaline and dilute acid-pretreated biomass (Figure 7). These glucose recoveries are in the upper part of the 64–95% saccharification observed for biomass with pretreatment and posterior alkaline delignification.^30,59^ The glucose yield was lower (around 60%) for miscanthus and bagasse and likely needed further process optimization for effective enzymatic hydrolysis (Figure 7), like the use of elevated pH to reduce the nonspecific cellulase binding to the lignin.^60^

The last aspect to take into account is the conservation of the native-like state of the lignin on all the tested biomasses (Table 2 and see Supporting Information Figure S9). The quantity of lignin retained in the residue of the ALACEN process varied (35–98%) depending on the biomass source. The corn-derived biomass is the most affected by the pretreatments, conserving under 50% of the initial biomass. Bagasse and miscanthus are the least affected, conserving more than 90% of the lignin. The same trend can be seen in the conservation of the lignin structure, where most of the biomasses show more than 40 per 100 aromatic units, while corn cobs and corn leaf present lower structure conservation (32 and 18, respectively), showing a correlation between structure conservation and mass retention in the final pellet. Lower amounts of β-O-4 were observed, indicating some structural degradation. However, no specific signals that indicate condensed aromatic structures in the form of condensed S units were observed by HSQC NMR for any of the residual lignin materials.^61^

## Conclusions

ALACEN is the first stepwise, flow-through fractionation approach with the capacity to obtain a fraction rich in esterified aromatics, a furfural-free monomeric xylose stream, a glucose fraction, and a native-like lignin-rich residue from herbaceous biomasses. The key feature of the ALACEN process is the xylose-rich stream obtained after the selective removal of esterified compounds. The monosaccharide nature of the xylose solubilized by the selected conditions and the absence of inhibitors in the dilute acid stream make it suitable for direct bacterial fermentation of bioplastics with similar yields as when commercial pure xylose is used in the fermentation. Furthermore, a high yield of glucose is obtained by enzymatic saccharification of the lignin-cellulose residue, which could be further converted to other valuable products. Finally, a lignin residue is cogenerated with a high conservation of the native β-O-4 structure, allowing for the obtention of 14% of aromatic monomers via selective depolymerization. In optimum conditions, the process can yield 80% FA removal, 51% of initial xylose as monosaccharides, 83% of initial glucan as glucose, and lignin with 55.3 β-O-4 motifs per 100 aromatic units. Furthermore, it is demonstrated that the process could be applied effectively to five other herbaceous biomasses, all providing xylose-rich streams suitable for PHA production. This opens the gate for further optimization of the process as well as for other applications of the obtained fractions. These will also need to be guided by life cycle assessment and technoeconomic analyses based on input and output products to further quantify the benefits of the ALACEN method.

## Supporting Information Available

Details about the setup, featuring technical diagrams and pictures; comprehensive insights into the detection and quantification of all the hydrogenolysis products, showcasing mass spectra and GC-FID results; further details on the characterization of PHA properties through TGA, DSC, and ^1^H NMR analyses; and characterization of lignin samples from various biomass sources by HSQC NMR (PDF)
