# Supplementary material for: ALACEN: A Holistic Herbaceous Biomass Fractionation Process Attaining a Xylose-Rich Stream for Direct Microbial Conversion to Bioplastics
Source: ACS Sustain Chem Eng. 2024 May 8;12(20):7724–38. doi: 10.1021/acssuschemeng.3c08414 (PMC11110678; doi:10.1021/acssuschemeng.3c08414)
Supplement: Supplementary file 1 — sc3c08414_si_001.pdf [file sc3c08414_si_001.pdf]

Electronic Supplementary Information

ALACEN: A holistic herbaceous biomass fractionation process attaining a xylose-rich stream for direct microbial conversion to bioplastics

Salvador Bertran-Llorens[a,ϒ], Wen Zhou[b,ϒ], Martín A. Palazzolo[a,c,d], Dana l. Colpa[b], Gert-Jan W. Euverink[b], Janneke Krooneman[b,e], Peter J. Deuss[a].*

a Green chemical reaction engineering, Engineering and Technology institute Groningen (ENTEG), University of Groningen, Nijenborgh 4, Groningen 9747 AG The Netherlands

b Products and processes for biotechnology, Engineering and Technology Institute Groningen (ENTEG), Faculty of Science and Engineering, University of Groningen, Nijenborgh 4, Groningen 9747 AG, The Netherlands

c Instituto Interdisciplinario de Ciencias Básicas (ICB, UNCuyo-CONICET) Padre Jorge Contreras 1300, Mendoza (5500), Argentina *(current address)*

d Instituto de Investigaciones en Tecnología Química (INTEQUI), FQByF, Universidad Nacional de San Luis, CONICET, Almirante Brown 1455, 5700 San Luis, Argentina

e Bioconversion and fermentation technology, Research Centre Biobased Economy, Hanze University of Applied Sciences, Zernikeplein 11, Groningen 9747 AS, The Netherlands

[ϒ] The authors contributed equally to this paper

* Corresponding author email: p.j.deuss@rug.nl

List of tables

Table S1: Total quantity (g) of each component during the ALACEN fractionation steps S14
Table S2 Comparison of water acid and base usage of a current commercial xylose production plant(Pang et al., 2021) and the ALACEN process. S23

List of figures

Figure S1: Piping and instrumentation diagram of the flow-through set up used for the ALACEN extraction. S4
Figure S2GC-FID chromatogram of the liquid fraction obtained after catalytic hydrogenolysis of the lignin obtained with the ALACEN process with wheat straw as initial biomass. S5
Figure S3: Mass spectra of the different products of the catalytic hydrogenolysis of lignin. S7
Figure S4: lignocellulose composition of wheat straw (initial) and dilute acid pretreated wheat straw (DDA). The dilute acid reaction for the DDA was conducted at 0.75w/w% of H_2SO_4 and 130°C.......... S8
Figure S5 Mass balance of the ALACEN process and the products obtained for 100g of initial wheat straw. S9
Figure S6: 1H NMR analysis of the bioplastics produced by S. thermodepolymerans with the diluted acid stream of the ALACEN fractionation under optimized conditions. S9
Figure S7: Thermal gravimetric analysis (TGA) analysis of the bioplastics produced by S. thermodepolymerans with the diluted acid stream of the ALACEN fractionation under optimized conditions.. S10
Figure S8 Differential scanning calorimetry (DSC) analysis of the bioplastics produced by S. thermodepolymerans with the diluted acid stream of the ALACEN fractionation under optimized conditions.. S11
Figure S9: Representation of the consumption over time of the total xylose (left) and PHB production over time (right) of S. thermodepolymerans with the stream obtained from the ALACEN process and the stream obtained with only a diluted acid reaction at the same conditions. S11
Figure S10: Gel permeation chromatography of the evolution of the direct diluted acid sample (green colors) and the xylose obtained after the ALACEN process (brown colors) during the bacterial production of PHA S12
Figure S11: Yield and identification of the monomeric phenolics obtained after the REL and ALACEN lignin catalytic hydrogenolysis. Amounts are corrected for quantity of lignin in the initial sample. To see the structure of the mentioned compounds refer to Supplementary Figure S2 and S3. .... S13
Figure S12. Gel-state NMR (4:1 $DMSO_{d6}$/$pyridine_{d5}$) spectrum of the residues after direct diluted acid and enzymatic saccharification in the flow-through system. S14
Figure S13: Gel-state NMR (4:1 $DMSO_{d6}$/$pyridine_{d5}$) spectrum of the residual enzymatic lignin. S17
Figure S14: Gel-state NMR (4:1 $DMSO_{d6}$/$pyridine_{d5}$) spectrum of the residue from wheat straw obtained after ALACEN process. S18

Figure S15. Gel-state NMR (4:1 $DMSO_{d6}$/$pyridine_{d5}$) spectrum of the residue from corn cobs obtained after ALACEN process. S19
Figure S16. Gel-state NMR (4:1 $DMSO_{d6}$/$pyridine_{d5}$) spectrum of the residue from bagasse obtained after ALACEN process. S20
Figure S17. Gel-state NMR (4:1 $DMSO_{d6}$/$pyridine_{d5}$) spectrum of the residue from miscanthus obtained after ALACEN process. S21
Figure S18. Gel-state NMR (4:1 $DMSO_{d6}$/$pyridine_{d5}$) spectrum of the residue from corn leaf obtained after ALACEN process. S22

Supplementary Notes

Supplementary Note S1: Green metrics evaluation 24

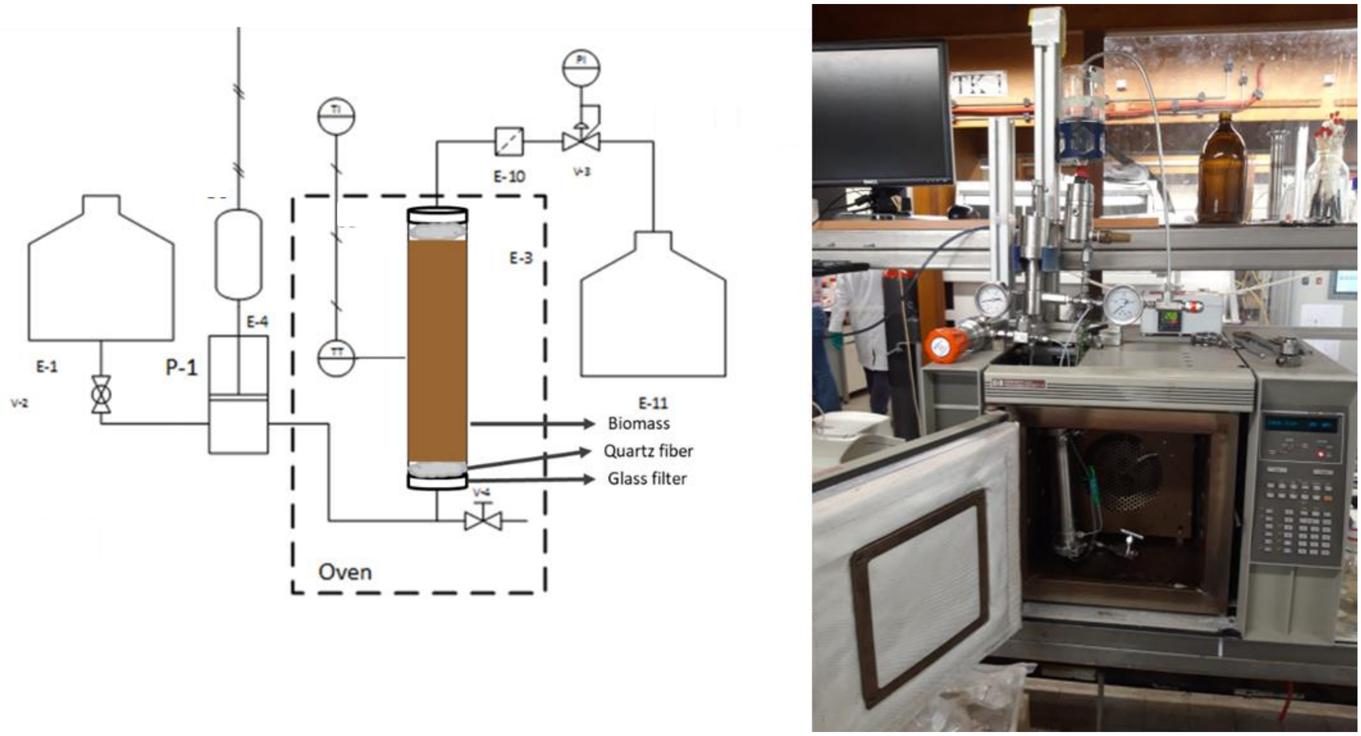

Tag	Description
E-1	Solvent tank
E-11	Extraction liquor collection tank
E-10	7-µm filter
E-3	100 mL reactor
PI	Pressure indicator
P-1	Reciprocating pump
V-2	Screw valve
V-3	Back-pressure valve
V-4	Screw valve
TI	Temperature indicator
TT	Temperature transmitter

Figure S1: Piping and instrumentation diagram of the flow-through set up used for the ALACEN extraction and a picture of the set-up.

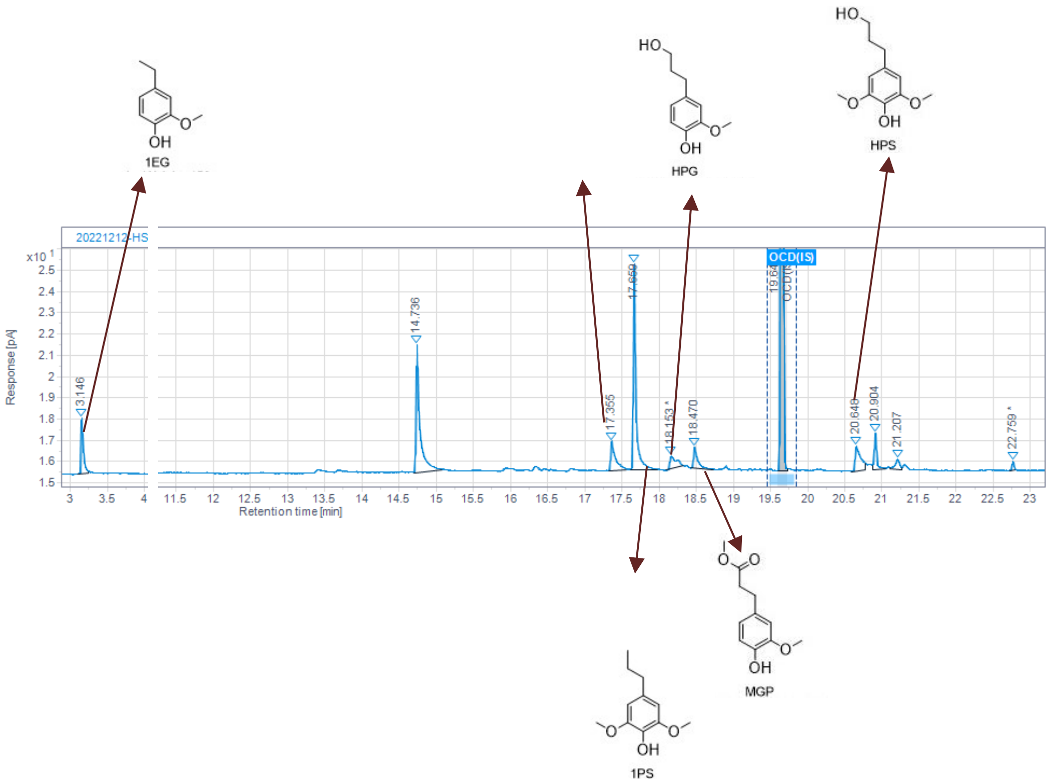

Figure S2GC-FID chromatogram of the liquid fraction obtained after catalytic hydrogenolysis of the lignin obtained with the ALACEN process with wheat straw as initial biomass.

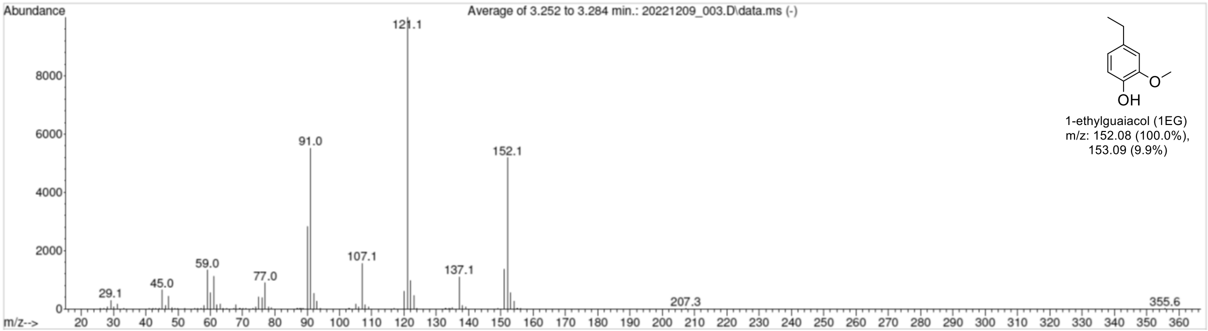
Abundance
Average of 3.252 to 3.284 min.: 20221209_003.D\data.ms (-)
m/z-->
29.1 45.0 59.0 77.0 91.0 107.1 121.1 137.1 152.1 207.3 355.6
OH
1-ethylguaiacol (1EG)
m/z: 152.08 (100.0%),
153.09 (9.9%)

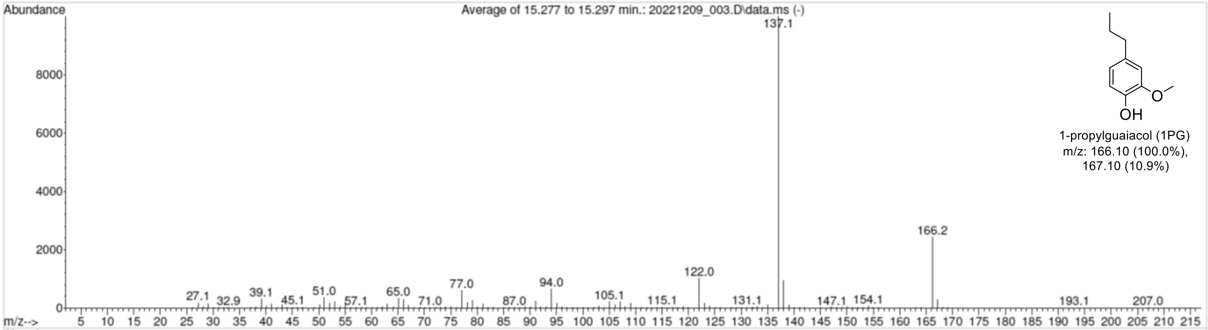
Abundance
Average of 15.277 to 15.297 min.: 20221209_003.D\data.ms (-)
m/z-->
27.1 32.9 39.1 45.1 51.0 57.1 65.0 71.0 77.0 87.0 94.0 105.1 115.1 122.0 131.1 137.1 147.1 154.1 166.2 193.1 207.0
OH
1-propylguaiacol (1PG)
m/z: 166.10 (100.0%),
167.10 (10.9%)

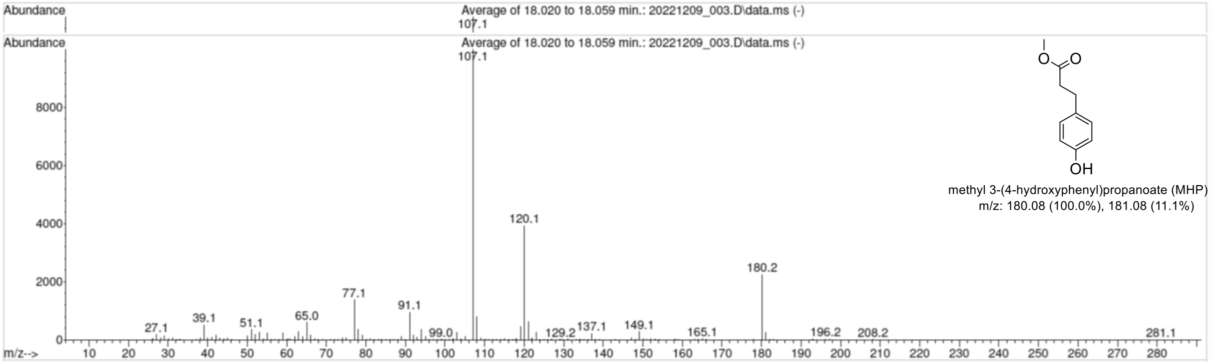
Abundance
Average of 18.020 to 18.059 min.: 20221209_003.D\data.ms (-)
m/z-->
27.1 39.1 51.1 65.0 77.1 91.1 99.0 107.1 120.1 129.2 137.1 149.1 165.1 180.2 196.2 208.2 281.1
OH
methyl 3-(4-hydroxyphenyl)propanoate (MHP)
m/z: 180.08 (100.0%), 181.08 (11.1%)

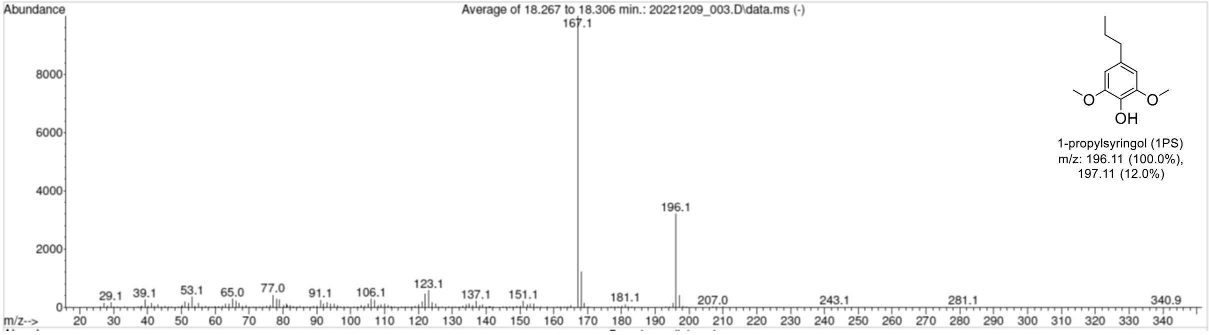
Abundance
Average of 18.267 to 18.306 min.: 20221209_003.D\data.ms (-)
m/z-->
29.1 39.1 53.1 65.0 77.0 91.1 106.1 123.1 137.1 151.1 167.1 181.1 196.1 207.0 243.1 281.1 340.9
OH
1-propylsyringol (1PS)
m/z: 196.11 (100.0%),
197.11 (12.0%)

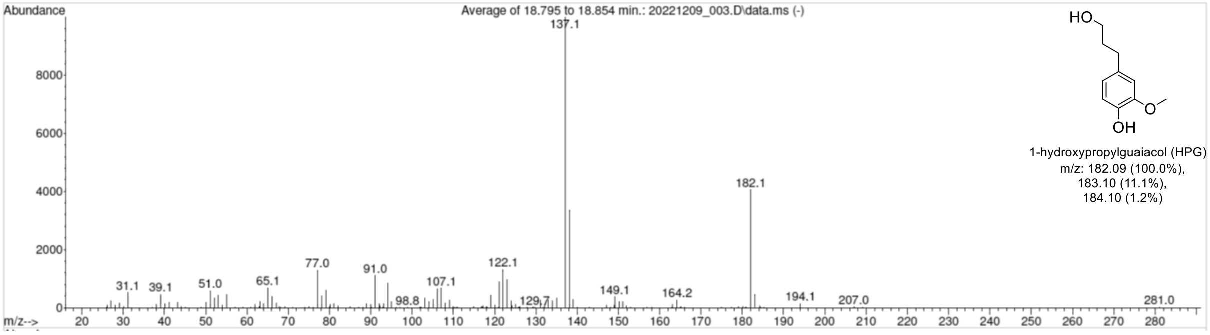
Abundance
Average of 18.795 to 18.854 min.: 20221209_003.D\data.ms (-)
137.1
182.1
31.1 39.1 51.0 65.1 77.0 91.0 98.8 107.1 122.1 129.7 149.1 164.2 194.1 207.0 281.0
m/z-->
1-hydroxypropylguaiacol (HPG)
m/z: 182.09 (100.0%),
183.10 (11.1%),
184.10 (1.2%)

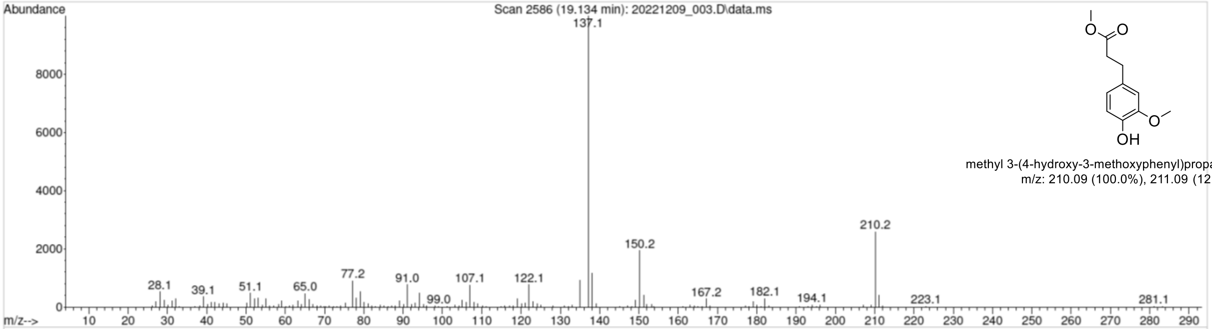
Abundance
Scan 2586 (19.134 min): 20221209_003.D\data.ms
137.1
210.2
150.2
28.1 39.1 51.1 65.0 77.2 91.0 99.0 107.1 122.1 167.2 182.1 194.1 223.1 281.1
m/z-->
methyl 3-(4-hydroxy-3-methoxyphenyl)propanoate (MGP)
m/z: 210.09 (100.0%), 211.09 (12.0%)

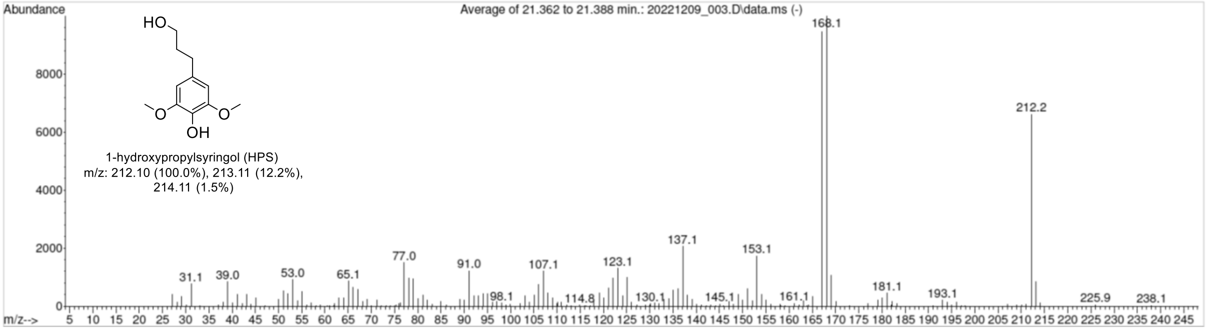
Abundance
Average of 21.362 to 21.388 min.: 20221209_003.D\data.ms (-)
168.1
212.2
31.1 39.0 53.0 65.1 77.0 91.0 98.1 107.1 114.8 123.1 130.1 137.1 145.1 153.1 161.1 181.1 193.1 225.9 238.1
m/z-->
1-hydroxypropylsyringol (HPS)
m/z: 212.10 (100.0%), 213.11 (12.2%),
214.11 (1.5%)

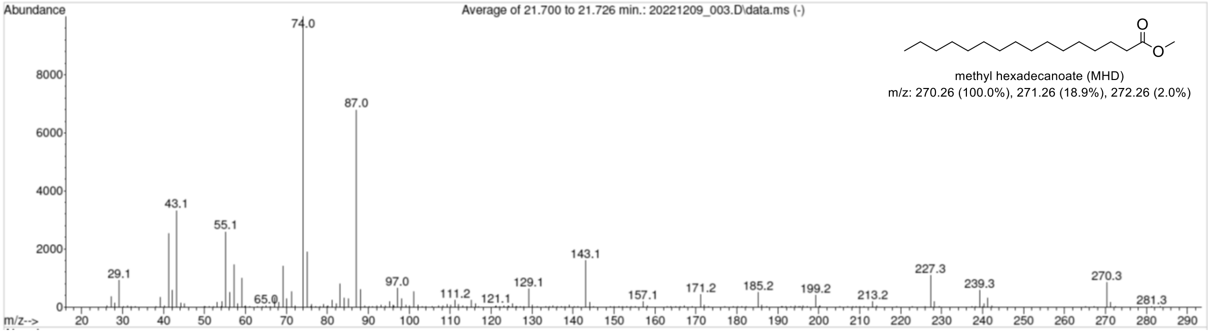
Abundance
Average of 21.700 to 21.726 min.: 20221209_003.D\data.ms (-)
74.0
87.0
29.1 43.1 55.1 65.0 97.0 111.2 121.1 129.1 143.1 157.1 171.2 185.2 199.2 213.2 227.3 239.3 270.3 281.3
m/z-->
methyl hexadecanoate (MHD)
m/z: 270.26 (100.0%), 271.26 (18.9%), 272.26 (2.0%)

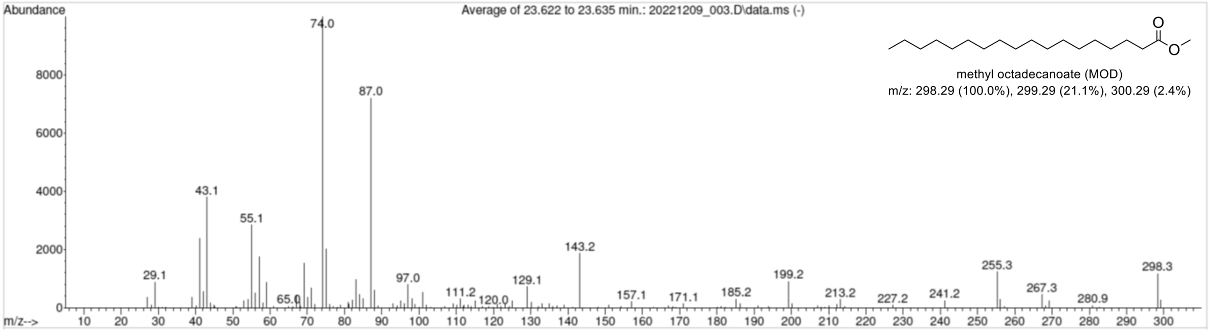

Figure S3: Mass spectra of the different products of the catalytic hydrogenolysis of lignin.

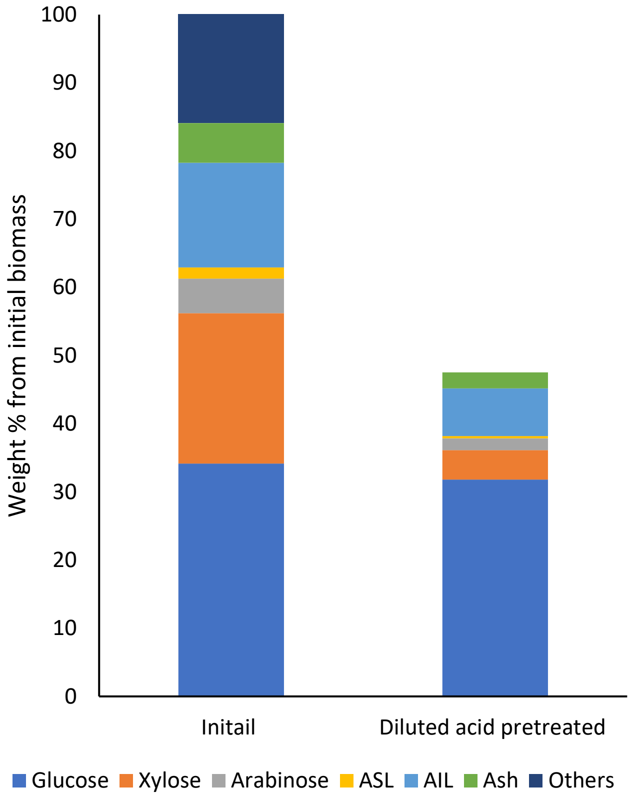

Figure S4: lignocellulose composition of wheat straw (initial) and dilute acid pretreated wheat straw (DDA). The dilute acid reaction for the DDA was conducted at 0.75w/w% of H_2SO_4 and 130°C.

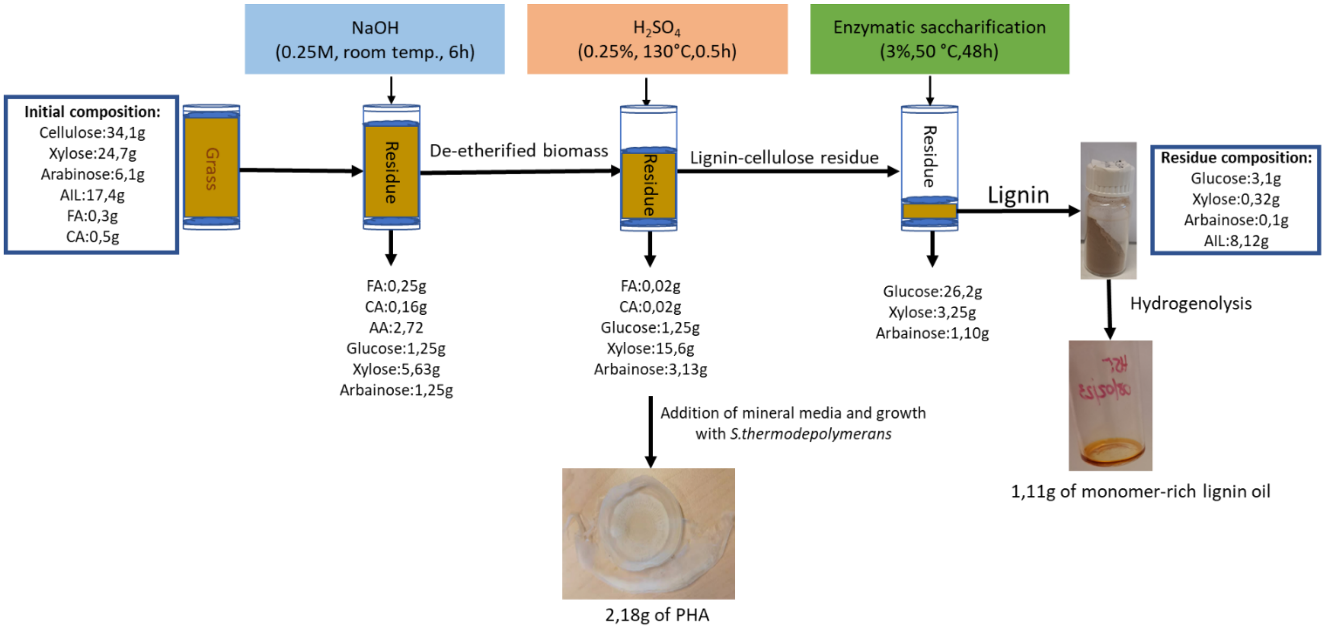

Figure S5 Mass balance of the ALACEN process and the products obtained for 100g of initial wheat straw.

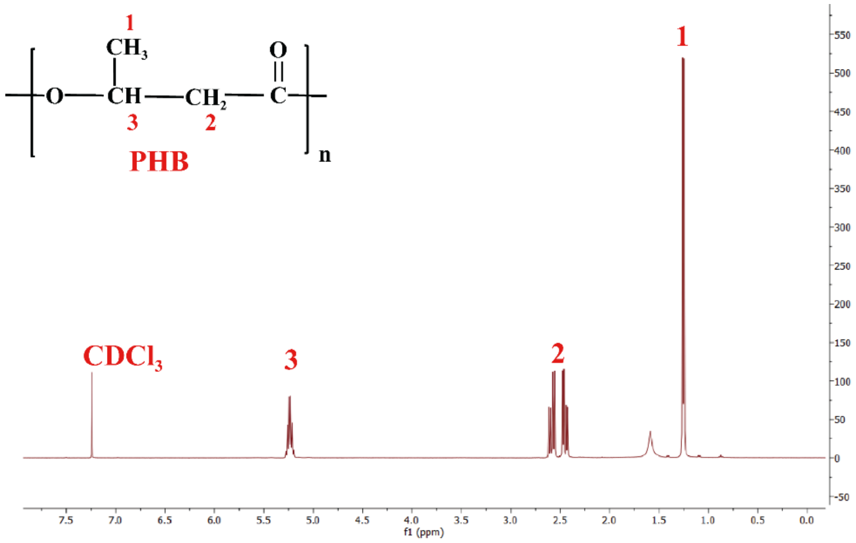

Figure S6: 1H NMR analysis of the bioplastics produced by S. thermodepolymerans with the diluted acid stream of the ALACEN fractionation under optimized conditions.

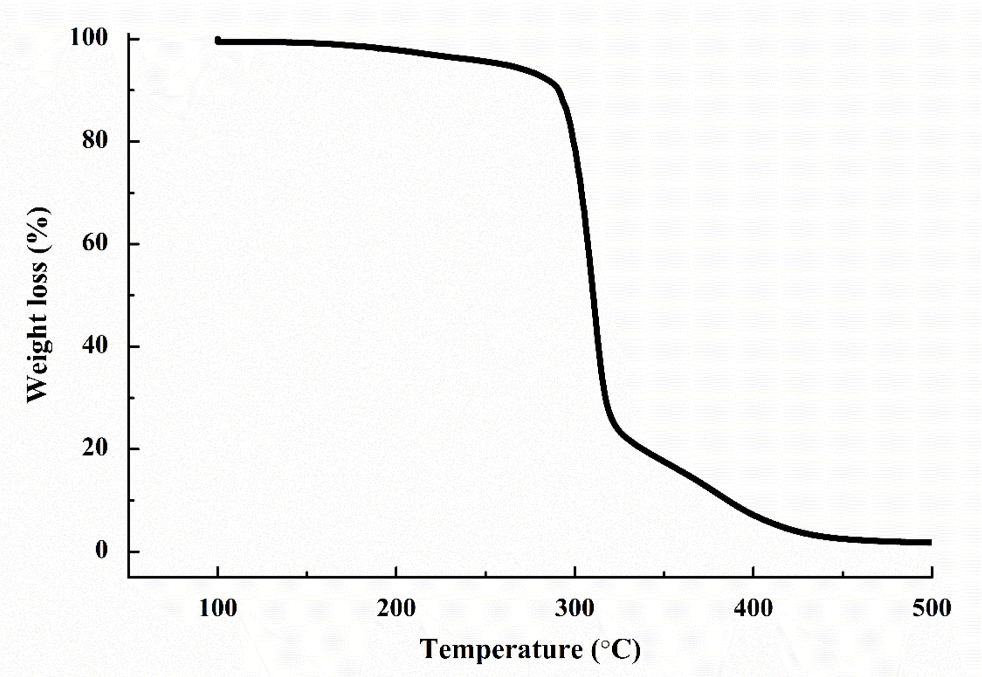

Figure S7: Thermal gravimetric analysis (TGA) analysis of the bioplastics produced by S. thermodepolymerans with the diluted acid stream of the ALACEN fractionation under optimized conditions.

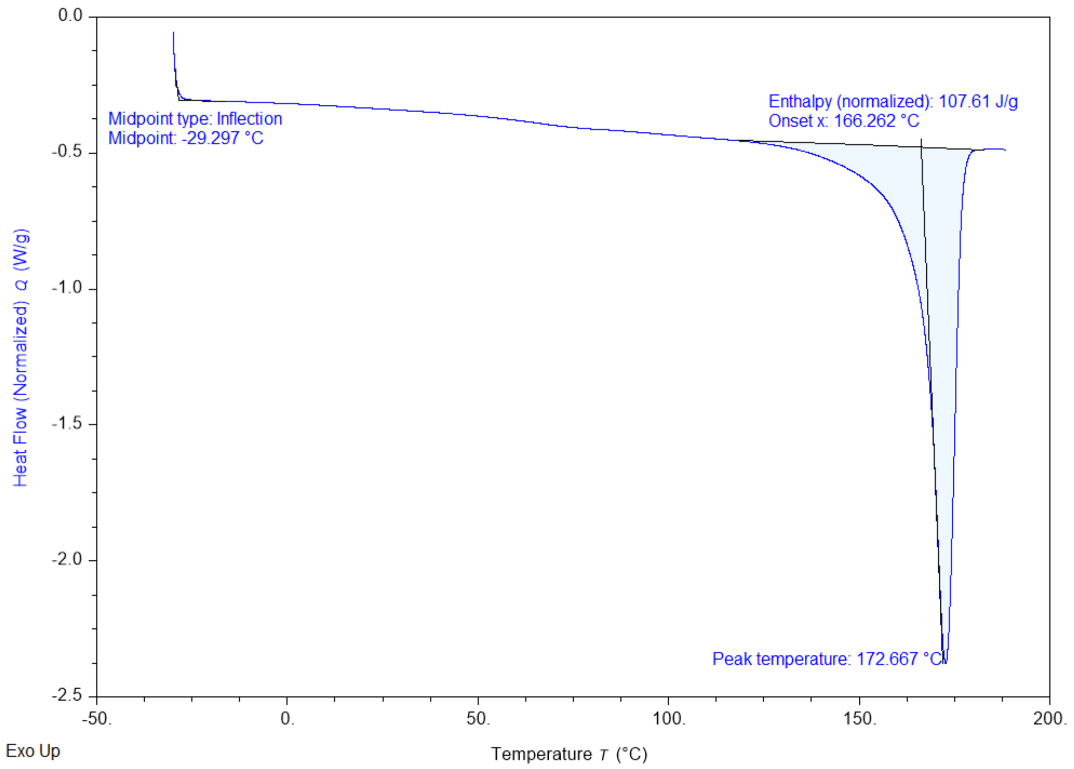

Figure S8: Differential scanning calorimetry (DSC) analysis of the bioplastics produced by S. thermodepolymerans with the diluted acid stream of the ALACEN fractionation under optimized conditions.

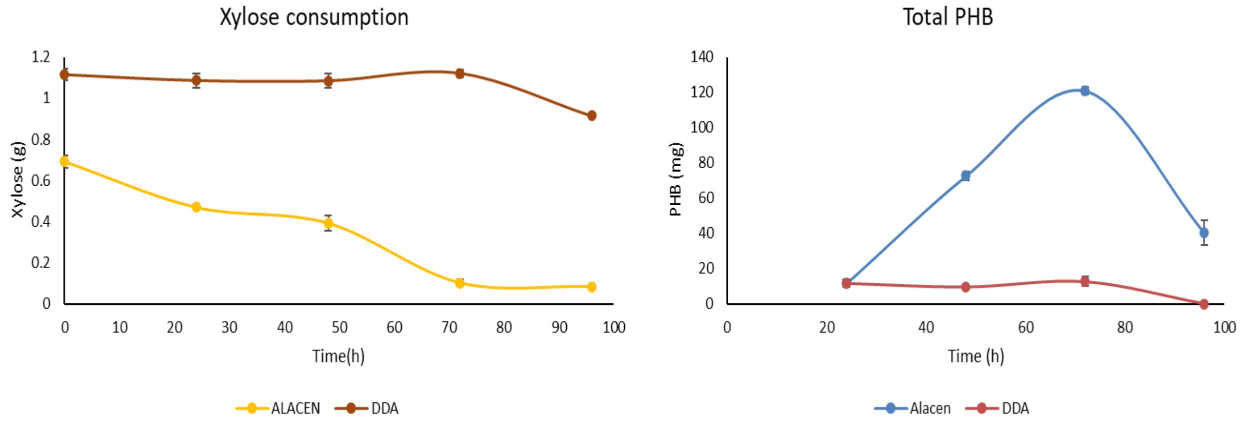

Figure S9: Representation of the consumption over time of the total xylose (left) and PHB production over time (right) of S. thermodepolymerans with the stream obtained from the ALACEN process and the stream obtained with only a diluted acid reaction at the same conditions.

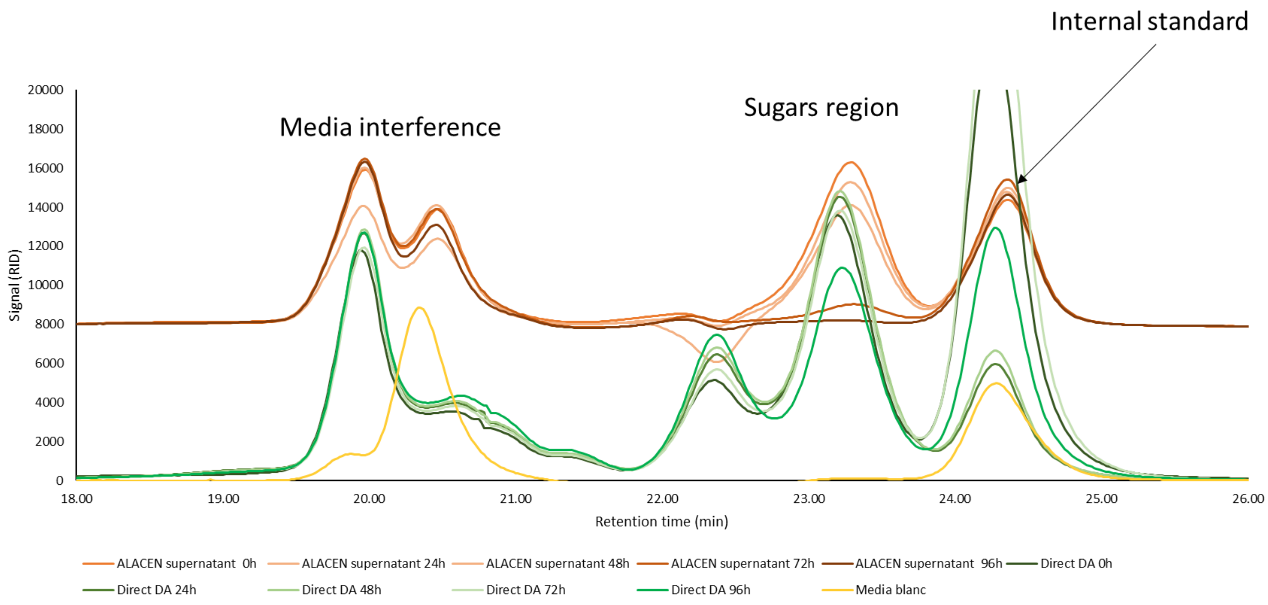

Figure S10: Gel permeation chromatography of the evolution of the direct diluted acid sample (green colors) and the xylose obtained after the ALACEN process (brown colors) during the bacterial production of PHA.

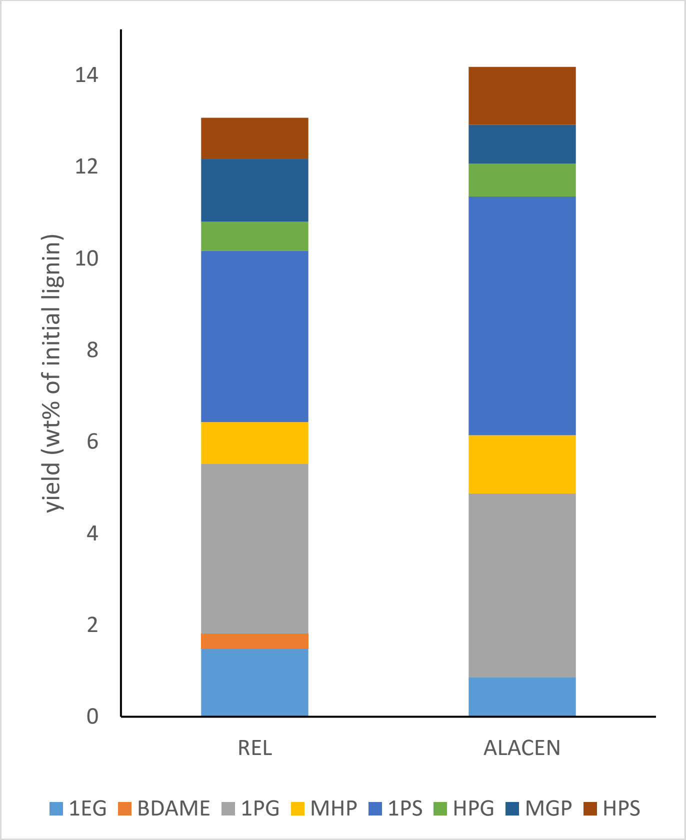

Figure S11: Yield and identification of the monomeric phenolics obtained after the REL and ALACEN lignin catalytic hydrogenolysis. Amounts are corrected for quantity of lignin in the initial sample. To see the structure of the mentioned compounds refer to Supplementary Figure S2 and S3.

Table S1: Total quantity (g) of each component during the ALACEN fractionation steps

		**Initial**	**AH**	**DA**	**ENZ**
**Wheat straw**	Cellulose	5.1	4.9	4.7	0.5
	Xylose	3.7	2.8	0.8	0.3
	Arabinose	0.9	0.7	0.2	0.2
	ASL	0.3	0.1	0.1	0.0
	AIL	2.3	1.8	1.5	1.3
	Weight	16.0	11.4	7.6	2.2
**Corn cobs**	Cellulose	5.9	5.5	5.1	0.2
	Xylose	5.4	4.8	0.7	0.1
	Arabinose	1.0	0.7	0.0	0.0
	ASL	0.5	-	-	0.0
	AIL	2.3	-	-	0.8
	Weight	16.0		6.8	1.3
**Bagasse**	Cellulose	4.2	4.1	3.9	1.3
	Xylose	2.4	1.9	0.0	0.0
	Arabinose	0.6	0.4	0.0	0.0
	ASL	0.2	-	-	0.0
	AIL	2.2	-	-	2.2
	Weight	10.0	-	7.0	3.6
**Reeds**	Cellulose	5.4	5.3	5.0	0.9
	Xylose	4.0	3.7	1.7	0.4
	Arabinose	1.2	1.2	0.7	0.0
	ASL	0.3	-	-	0.00
	AIL	3.5	-	-	2.6
	Weight	16.0	-	8.5	3.9
**Olifant**	Cellulose	5.8	5.7	5.6	2.1

	Xylose	3.1	3.0	1.2	0.5
	Arabinose	0.9	0.9	0.6	0.0
	ASL	0.2	-	-	0.0
	AIL	3.0	-	-	2.9
	Weight	13.0	-	7.9	4.9
**Corn leaf**	Cellulose	5.7	5.7	5.4	0.3
	Xylose	4.9	4.7	1.6	0.1
	Arabinose	1.3	1.2	0.5	0.0
	ASL	0.3	-	-	0.0
	AIL	1.5	-	-	0.7
	Weight	15.0	-	6.7	1.5
**Beechwood**	Cellulose	5.9	5.9	5.8	2.9
	Xylose	2.8	2.6	1.3	0.8
	Arabinose	1.1	1.1	0.9	0.0
	ASL	0.2	-	-	0.0
	AIL	5.0	-	-	2.6
	Weight	16.0	-	11.2	8.0
**wheat straw direct DA**	Cellulose	5.1	5.1	4.6	1.3
	Xylose	3.7	3.7	1.6	0.6
	Arabinose	0.9	0.9	0.5	0.0
	ASL	0.3	0.3	0.0	0.0
	AIL	2.3	2.3	2.0	1.8
	Weight	16.0	16.0	7.5	3.7

A) the NREL protocol was performed after each stage of the ALACEN process, b) NREL was performed only for the initial biomass and the residue after the ALACEN process, for the rest of the stages acid hydrolysis with 4%w/w H_2SO_4 at 120°C was performed on the supernatant, and therefore the lignin content is missing in the mild alkaline (AH) and diluted acid (DA) treatments, indicated by -

Direct diluted acid

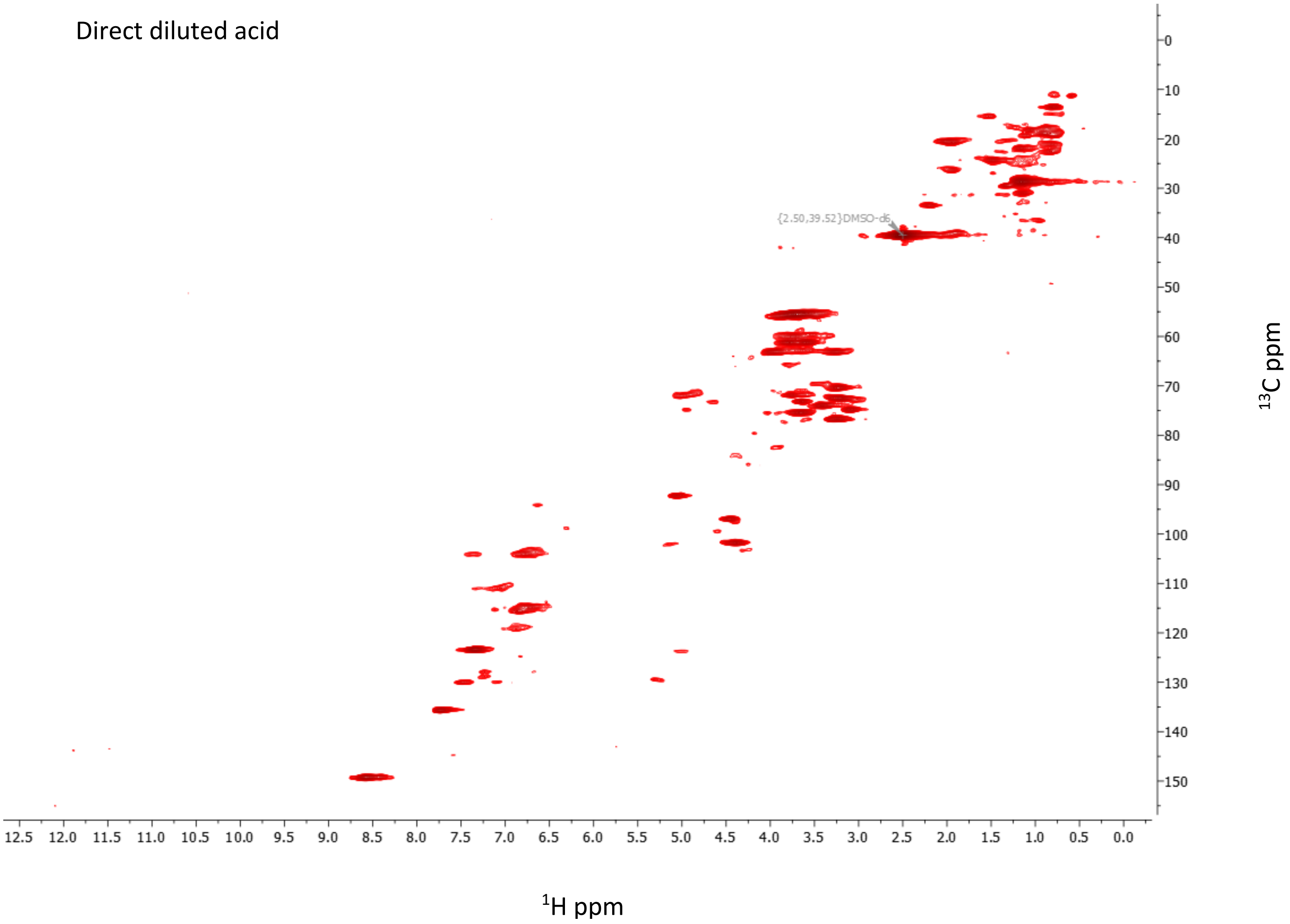

Figure S12. Gel-state NMR (4:1 $DMSO_{d6}$/$pyridine_{d5}$) spectrum of the residues after direct diluted acid and enzymatic saccharification in the flow-through system.

Residual enzymatic lignin

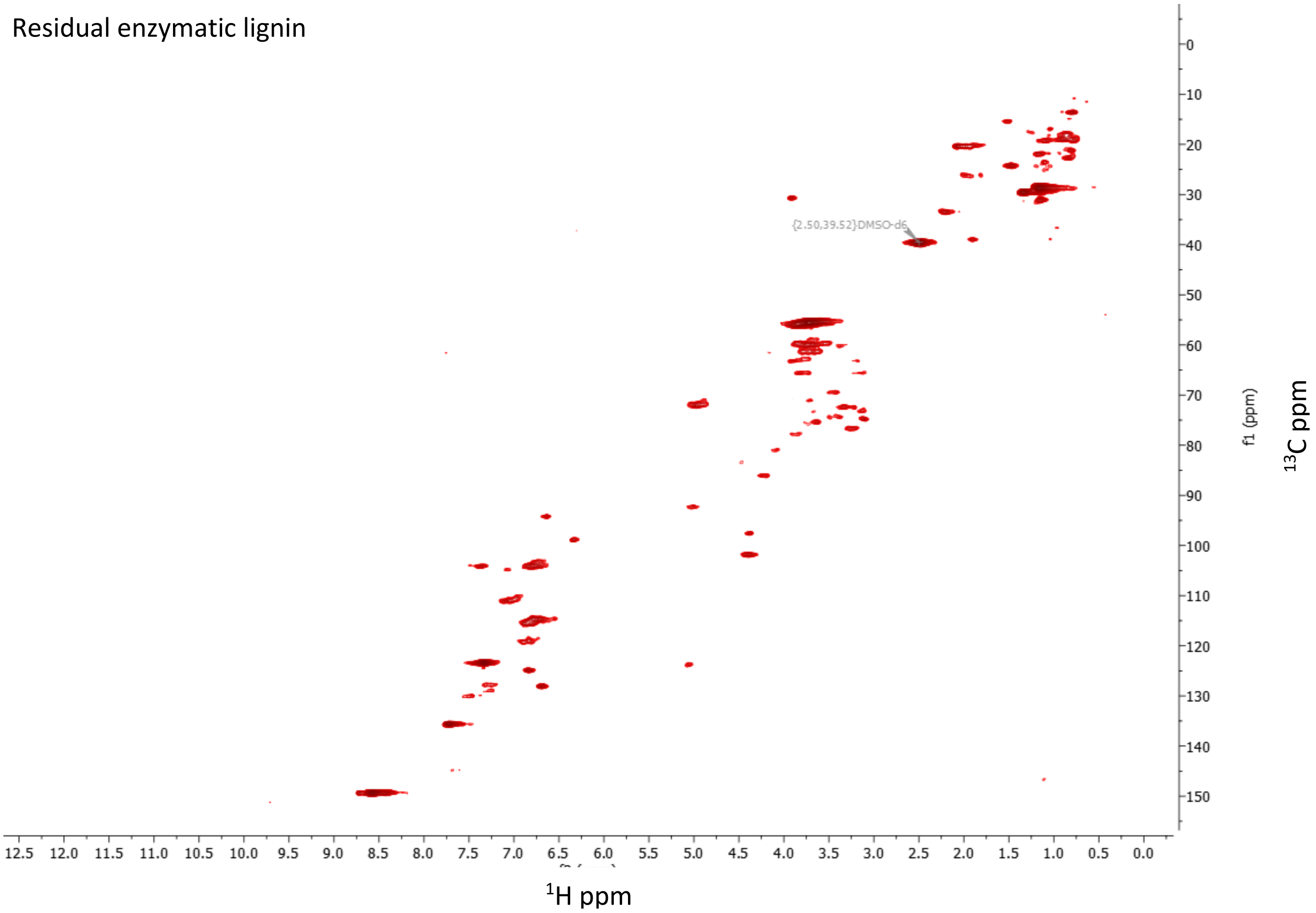

Figure S13: Gel-state NMR (4:1 $DMSO_{d6}$/$pyridine_{d5}$) spectrum of the residual enzymatic lignin.

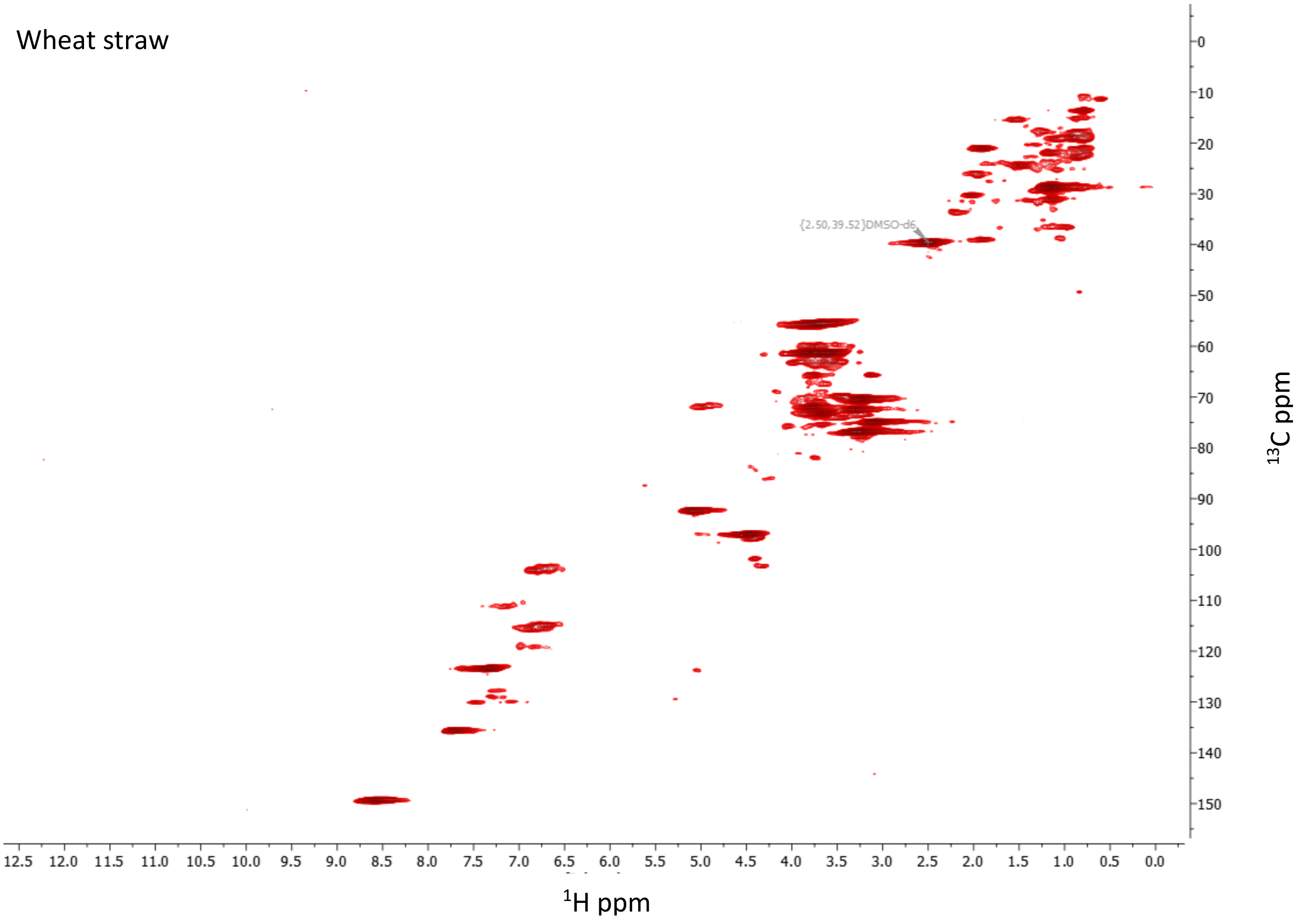

Figure S14: Gel-state NMR (4:1 $DMSO_{d6}$/$pyridine_{d5}$) spectrum of the residue from wheat straw obtained after ALACEN process.

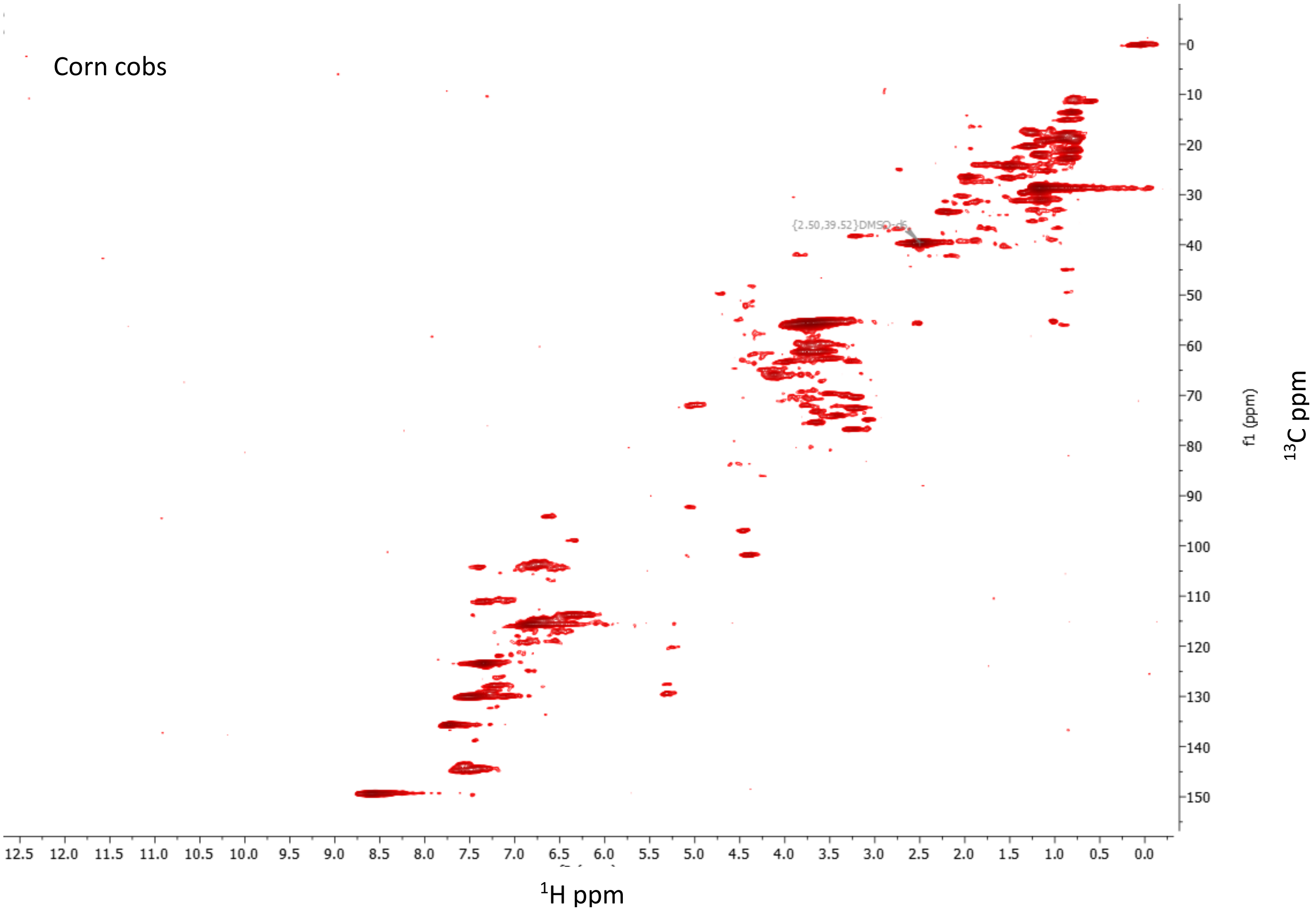

Figure S15. Gel-state NMR (4:1 $DMSO_{d6}$/$pyridine_{d5}$) spectrum of the residue from corn cobs obtained after ALACEN process.

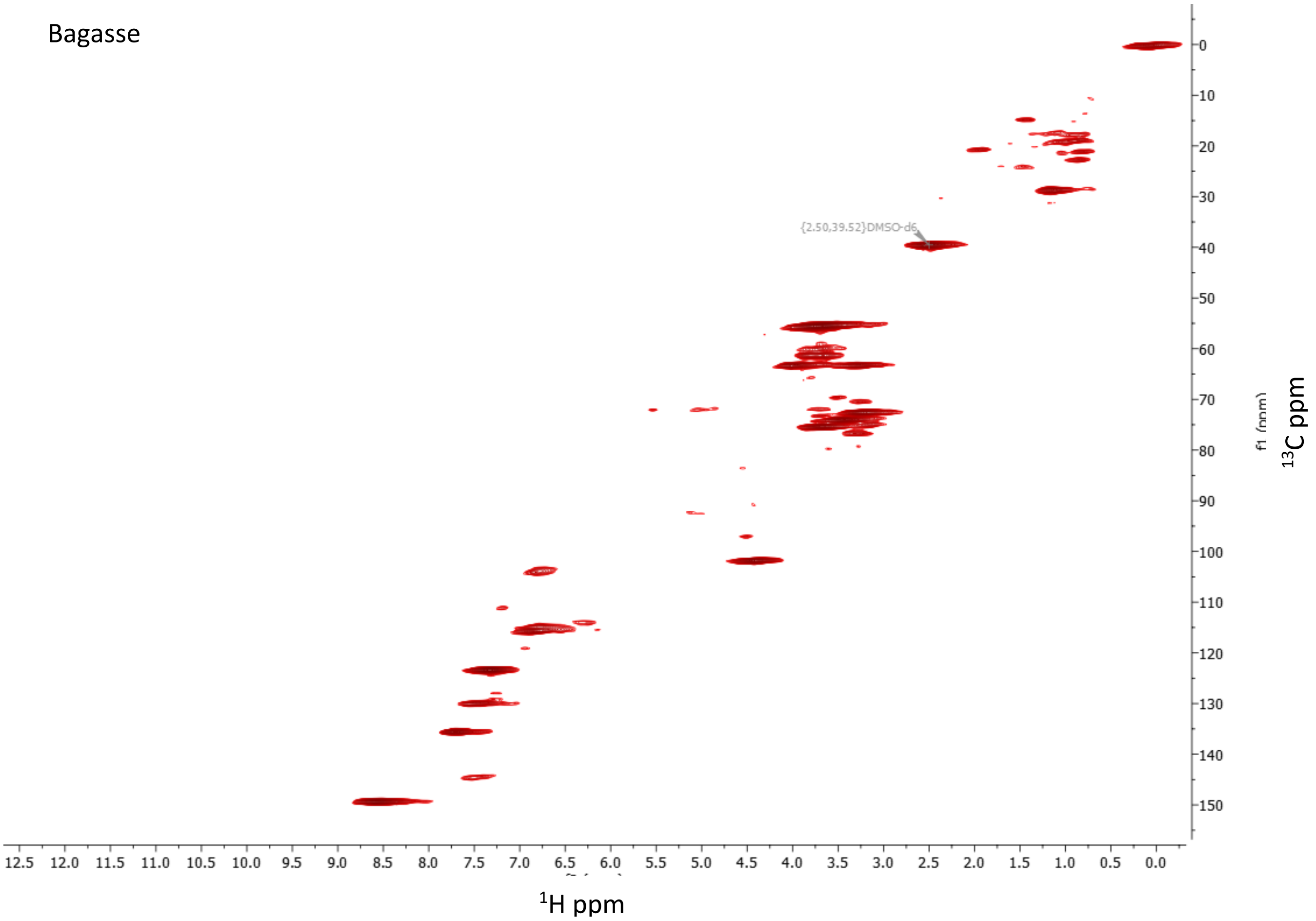

Figure S16. Gel-state NMR (4:1 $DMSO_{d6}$/$pyridine_{d5}$) spectrum of the residue from bagasse obtained after ALACEN process.

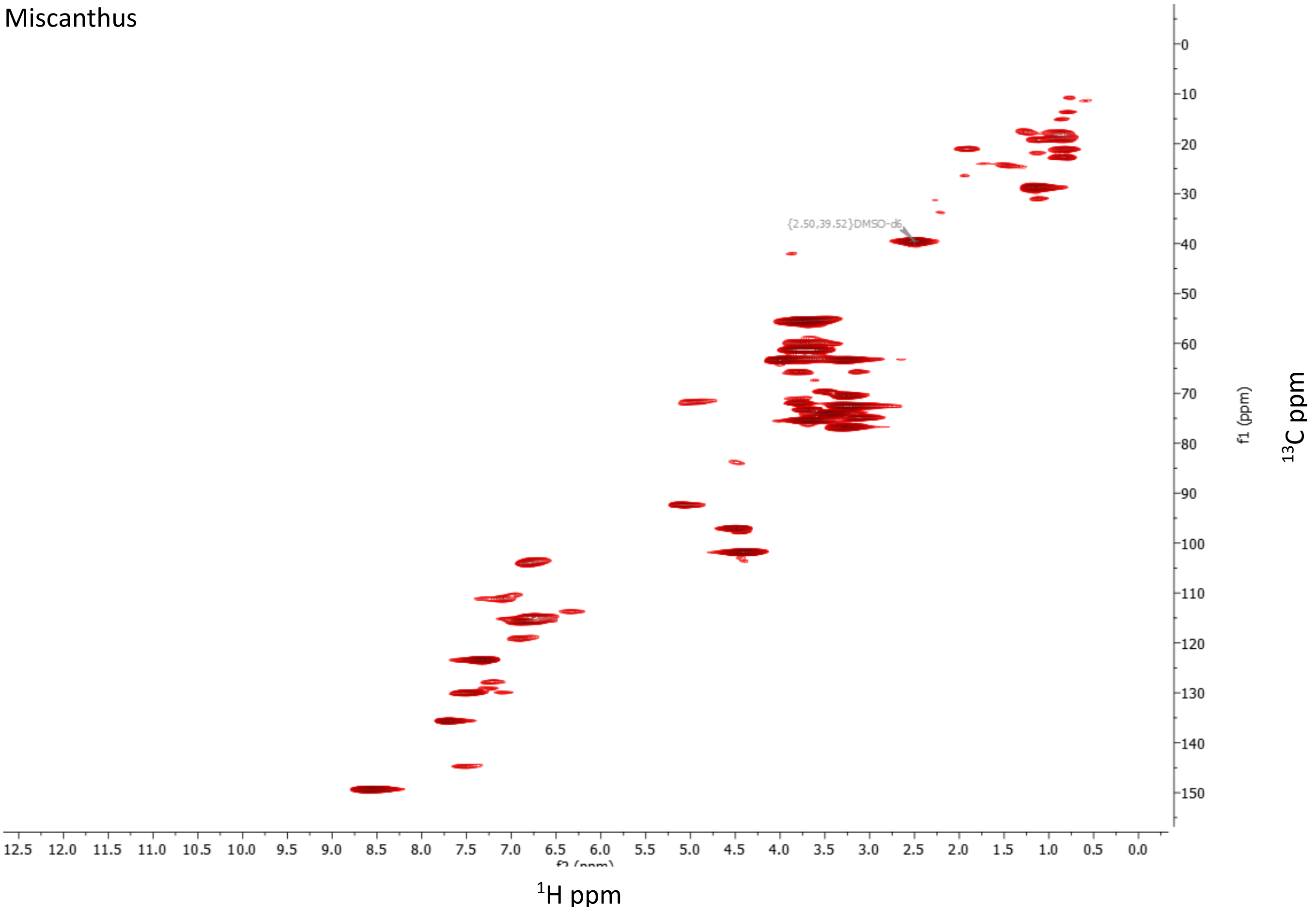

Figure S17. Gel-state NMR (4:1 $DMSO_{d6}$/$pyridine_{d5}$) spectrum of the residue from miscanthus obtained after ALACEN process.

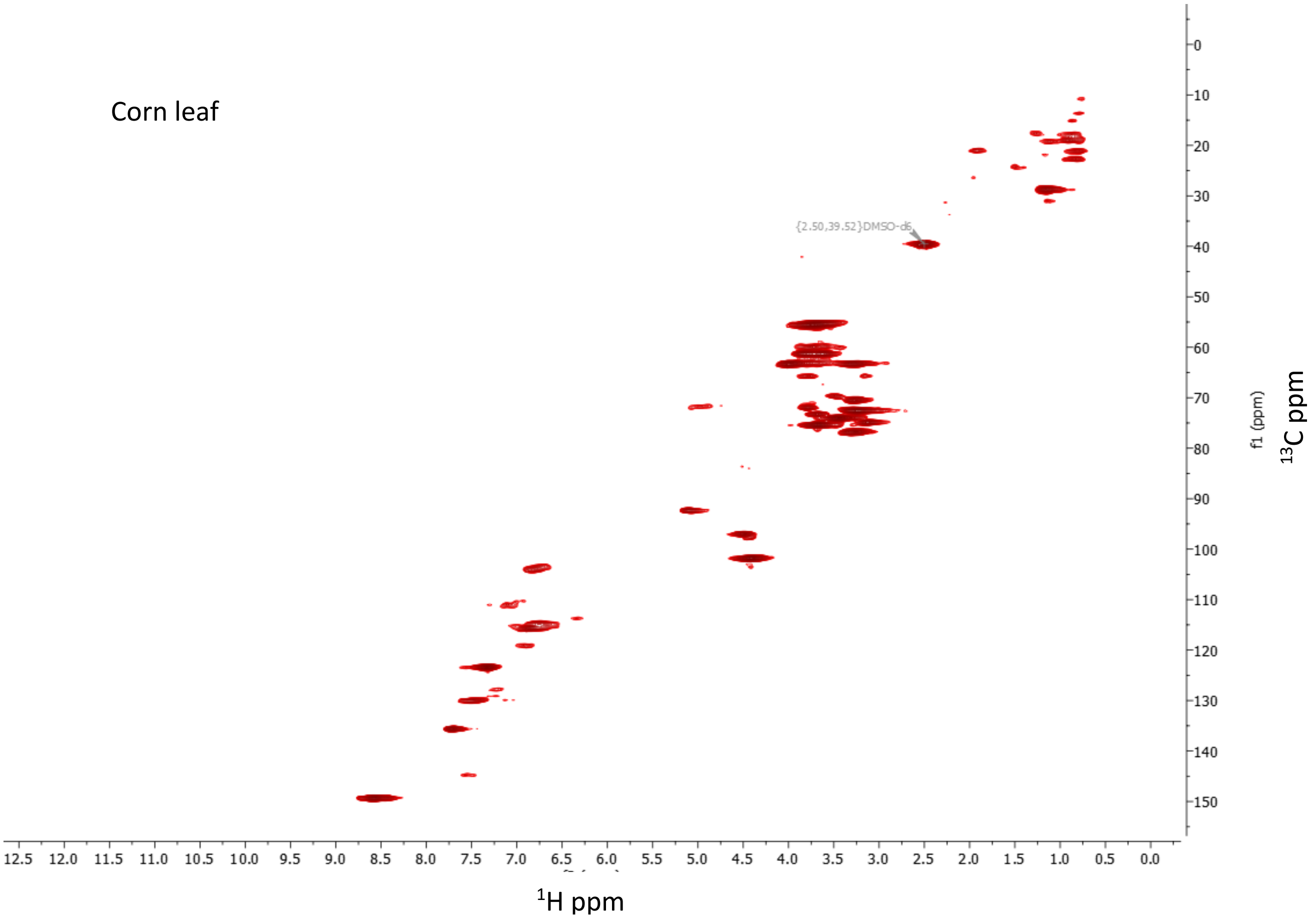

Figure S18. Gel-state NMR (4:1 $DMSO_{d6}$/$pyridine_{d5}$) spectrum of the residue from corn leaf obtained after ALACEN process.

Table S2 Comparison of water acid and base usage of a current commercial xylose production plant[1] and the ALACEN process.

Current commercial process

	(water tonne/tonne biomass)	Acid used (kg/tonne)	Base used (kg/tonne)	E-factor
**Pretreatment**	5.70	3.81		
**Hydrolysis**	2.13	34.29		
**Total purification**	18.57	0.14	0.14	
**Alkaline**	5.70		0.30	
**Llignin purification**		0.09		
**solid washing**	2.80	0.01		
**cellulose rich residue conversion**	2.91			
**Use for tone of biomass**	34.91	38.33	0.44	

ALACEN process

	(water tonne/tonne biomass)	Acid used (kg/tonne)	Base used (kg/tonne)	E-factor
**Conditioning (filling colum)**	6.25		0.0625	
**Diluted alkali**	13		0.13	
**Washing**	31.25			
**Conditioning (filling colum)**	6.25	0.046875		
**Diluted acid**	12	9		
**Washing**	31.25			
**Cellulose conversion**	31.25			
**Use for tone of biomass**	131.25	9.05	0.19	
**% from the current process**	376.0	23.6	43.5	

Supplementary Note S1: Green metrics evaluation

The green metrics evaluation for the PHA production from the hemicellulose streams was done according to previous publication [2]. For the evaluation of the ALACEN and the commercial process decried in [1], the steps to obtain fermentable monomeric xylose were accounted and the PHA yields for pure xylose and the ALACEN stream were used. For ALACEN process, the diluted alkali and diluted acid were taken into account while for the commercial process only the diluted acid and the purification to obtain monomeric xylose were accounted. Two E-values are evaluated, one including water usage and one only evaluating chemicals as waste. This is because the ALACEN process still needs further optimization and the water reuse strategies can be implemented, therefore for a proper evaluation of the green metrics the two values are shown. Moreover, this metric does not evaluate the fact that ALACEN process also obtains a hydroxamic acids rich streams, and that at this point the biomass is ready to be enzymatically hydrolyzed to glucose, while the commercial process still needs a alkaline pretreatment to achieve the same saccharification levels.

The equation to measure the E-value was:

$$\mathrm{E-factor} = \frac{\mathrm{Mass\ of\ wastes}}{mass\ of\ product}$$

	Commercial process	ALACEN
**Xylose production (Ton/Ton of biomass)**	0.14	0.14
**PHA production (Kg/Ton of biomass)**	22.06	19.21

**Waste generation with water Ton / Ton of biomass**	26.44	68.75
**Waste generation with no water Ton / Ton of biomass**	0.04	0.01
**E-factor with water**	1198.30	3578.64
**E-factor without water**	1.76	0.48

References

(1) Pang, B.; Sun, Z.; Wang, L.; Chen, W. J.; Sun, Q.; Cao, X. F.; Shen, X. J.; Xiao, L.; Yan, J. L.; Deuss, P. J.; Yuan, T. Q.; Sun, R. C. Improved Value and Carbon Footprint by Complete Utilization of Corncob Lignocellulose. *Chem. Eng. J.* **2021**, *419*, 129565. https://doi.org/10.1016/J.CEJ.2021.129565.

(2) Matos, C. T.; Gouveia, L.; Morais, A. R. C.; Reis, A.; Bogel-ŁUkasik, R. Green Metrics Evaluation of Isoprene Production by Microalgae and Bacteria. *Green Chem.* **2013**, *15* (10), 2854–2864. https://doi.org/10.1039/C3GC40997J.
